# Ultrasound-Based Radiomics Analysis for Predicting Disease-Free Survival of Invasive Breast Cancer

**DOI:** 10.3389/fonc.2021.621993

**Published:** 2021-04-29

**Authors:** Lang Xiong, Haolin Chen, Xiaofeng Tang, Biyun Chen, Xinhua Jiang, Lizhi Liu, Yanqiu Feng, Longzhong Liu, Li Li

**Affiliations:** ^1^ Department of Medical Imaging, Collaborative Innovation Center for Cancer Medicine, State Key Laboratory of Oncology in South China, Sun Yat-Sen University Cancer Center, Guangzhou, China; ^2^ School of Biomedical Engineering, Southern Medical University, Guangzhou, China; ^3^ Guangdong Provincial Key Laboratory of Medical Image Processing, Southern Medical University, Guangzhou, China; ^4^ Guangdong-Hong Kong-Macao Greater Bay Area Center for Brain Science and Brain-Inspired Intelligence, Southern Medical University, Guangzhou, China; ^5^ Department of Ultrasound, Collaborative Innovation Center for Cancer Medicine, State Key Laboratory of Oncology in South China, Sun Yat-Sen University Cancer Center, Guangzhou, China

**Keywords:** breast cancer, radiomics, ultrasound, disease-free survival, nomogram

## Abstract

**Background:**

Accurate prediction of recurrence is crucial for personalized treatment in breast cancer, and whether the radiomics features of ultrasound (US) could be used to predict recurrence of breast cancer is still uncertain. Here, we developed a radiomics signature based on preoperative US to predict disease-free survival (DFS) in patients with invasive breast cancer and assess its additional value to the clinicopathological predictors for individualized DFS prediction.

**Methods:**

We identified 620 patients with invasive breast cancer and randomly divided them into the training (n = 372) and validation (n = 248) cohorts. A radiomics signature was constructed using least absolute shrinkage and selection operator (LASSO) Cox regression in the training cohort and validated in the validation cohort. Univariate and multivariate Cox proportional hazards model and Kaplan–Meier survival analysis were used to determine the association of the radiomics signature and clinicopathological variables with DFS. To evaluate the additional value of the radiomics signature for DFS prediction, a radiomics nomogram combining the radiomics signature and clinicopathological predictors was constructed and assessed in terms of discrimination, calibration, reclassification, and clinical usefulness.

**Results:**

The radiomics signature was significantly associated with DFS, independent of the clinicopathological predictors. The radiomics nomogram performed better than the clinicopathological nomogram (C-index, 0.796 *vs.* 0.761) and provided better calibration and positive net reclassification improvement (0.147, *P *= 0.035) in the validation cohort. Decision curve analysis also demonstrated that the radiomics nomogram was clinically useful.

**Conclusion:**

US radiomics signature is a potential imaging biomarker for risk stratification of DFS in invasive breast cancer, and US-based radiomics nomogram improved accuracy of DFS prediction.

## Introduction

Recurrence remains the principal cause of breast cancer-related death, which seriously endanger the health of women (1, 2). More intensive therapy seems to improve prognosis for patients at high risk of recurrence (3). For predicting breast cancer recurrence, many prognostic models have been developed based on the clinicopathological factors like tumor size, nodal status, and Ki-67 expression, but the performance of most models declined for some independent populations (4). Gene tests have been reported to predict patient outcome (5), but they are difficult to be widely used in clinically due to the high price and complex operation. More convenient and appropriate methods to enhance recurrence prediction for breast cancer is the need of the hour.

Radiomics holds promise in predicting breast cancer recurrence due to its high-dimensional features extracted from medical images (6), which are not only related to the multigene assay recurrence scores of breast cancer but also associated to the recurrence survival (7–9). However, most previous studies about radiomics and breast cancer survival conducted thus far were based on magnetic resonance imaging (MRI). Ultrasound (US) is a safe, inexpensive, and widely available modality. US radiomics features could distinguish benign breast tumors from malignant tumors, could predict axillary lymph node metastasis, and could assist clinicians with accurate prognosis prediction in breast cancer (10–12). Therefore, whether US radiomics features could be used to predict breast cancer recurrence is merits further investigation.

Considering the above findings, a multiple-feature-based radiomics signature extracted from preoperative US images was developed for predicting disease-free survival (DFS) of invasive breast cancer in our study and its additional value added to the clinicopathological predictor was further assessed.

## Materials and Methods

### Patients

This study has obtained the ethical approval from the institutional review board, the informed patient consent was waived due to the nature of retrospective analysis. From February 2014 to November 2016, 812 consecutive women of breast cancer were identified. The inclusion criteria included: (1) patients with complete clinicopathological data and follow-up information; (2) primary unilateral invasive breast cancer confirmed by histopathology; (3) US examination performed within 2 weeks preoperatively (4); patients with no anticancer therapy before US examination; and (5) patients without history of breast cancer and/or other malignancy. The exclusion criteria included: (1) patients who received preoperative neoadjuvant chemotherapy; (2) patients presenting with metastatic disease; (3) insufficient quality of images and/or only partial tumor included in the images; and (4) patients lost to follow up. Finally, we enrolled 620 patients (mean age: 49.62 years, range: 27–87 years) (**Figure S1**) and divided them into the training cohort (n = 372) and validation cohort (n=248) randomly.

### Clinicopathological Data

Medical records were reviewed to acquire the clinical and pathological data, including: age; status of menopausal; history of risk factors for breast cancer (including family history of breast cancer and/or benign breast disease history); surgery type and adjuvant treatment (radiotherapy, chemotherapy, endocrine therapy, targeted therapy); pathologic tumor size; histologic type; TNM stage; T stage; N stage; lymphovascular invasion (LVI); invasion of nerves; associated ductal carcinoma *in situ* (DCIS); and status of estrogen receptor (ER), progesterone receptor (PR), human epidermal growth factor receptor 2 (HER2), and Ki-67, which were assessed by immunohistochemistry (IHC) and fluorescence *in situ hybridization* (FISH). ER/PR was defined as positive if nuclear staining was present in ≥1% cells (13). The HER2 status was scored as 0, 1+, 2+, or 3+. Scores 0 and 1+ were defined as negative, and score 3+ as positive. Score 2+ was considered indeterminate and was further confirmed with FISH (14). According to results of IHC and FISH, tumors were categorized into the following four subtypes: luminal A, luminal B, HER2-enriched and triple-negative (15). The targeted therapy was anti-HER2 therapy using trastuzumab. The American Joint Committee on Cancer TNM Staging Manual, 7th edition (16), was used for tumor stage.

### Follow-Up

DFS was considered as the end point of the present study, which was defined as the interval time between the surgery and recurrence or breast cancer-related death, whichever came first. Recurrence means locoregional recurrence, distant metastasis, or contralateral breast cancer (17). Physical examination, histopathology, and imaging modalities such as US, computed tomography, MRI were used to demonstrated the recurrence. At the last follow-up, patients without an event and/or died of non-breast cancer related events were censored; two patients died from cardiovascular disease in this study.

### Imaging Acquisition, Radiomics Analysis and Radiomics Signature Construction

**Figure S2** shows the radiomics workflow. US images were collected in different machines (**Table S1**) and exported from the data system of our hospital. Radiologist 1 (6 years’ experience) selected one greyscale image with the largest cross-section for every breast tumor, and drew a single region-of-interest (ROI) along the tumor margin by Photoshop software (**Figure S3**). Then, the ROIs were validated by radiologist 2 (10 years’ experience). The radiologists did not know the results of pathology. For multifocal (MF) or multicentric (MC) disease (18), we chose the largest tumor to analysis. After the ROIs were defined, radiomics features which could be divided into four categories, including first-order statistics features, two-dimensional (2D) shape-based features, texture features, and wavelet features, were extracted using the “PyRadiomics” package in Python software (19). Then, a two-step feature selection method which comprised by Sperman correlation coefficients and Ward linkage method, and least absolute shrinkage and selection operator (LASSO) Cox method were performed (20, 21). Finally, a radiomics signature was constructed, and a radiomics score (Rad-score) was calculated at the same time. In the supplementary materials, there are more details.

The intra-observer agreement of feature extraction was evaluated by inter-class correlation coefficient (ICC). We randomly selected 95 patients and redrew ROIs by radiologist 1 one month later after the first ROI segmentation. An ICC >0.75 indicated a good reproducibility.

### Validation of Radiomics Signature

In order to assess the association of the radiomics signature with DFS, patients were divided into a high risk and a low risk groups using the cutoff of the Rad-score identified by X-tile (22). We performed Kaplan–Meier survival analysis to analyze DFS between these two groups and the differences of survival curves were determined by Log-rank tests. We also assessed the association of the single selected feature with DFS by the same way. Then, distribution of Rad-score and DFS along with the selected features’ expression were assessed. Stratified analyses were performed using subgroups within the molecular subtype and categorical clinicopathological variables.

The univariate Cox proportional hazards model was used to analyze the effects of the clinicopathological variables and radiomics signature on DFS. Then, the most useful predictors were selected using multivariate Cox proportional hazards model by including clinicopathological variables in a step-wise (forward and backward) manner based on the Bayesian information criterion (BIC). Finally, the radiomics signature was integrated into a multivariable Cox proportional hazards model to evaluate its performance in DFS prediction.

All the above analyses were first performed in the training cohort, and then validated in the validation cohort, except for the stratified analyses which were performed in the whole cohort.

### The Additional Value of Radiomics Signature for DFS Prediction

In order to evaluate the additional value of the radiomics signature for DFS prediction, a radiomics nomogram containing the radiomics signature and clinicopathological predictors was constructed and compared with a clinicopathological nomogram containing only the clinicopathological predictors. The performance of the nomogram was assessed in the following four aspects: (1) discrimination, it was evaluated by Harrell’s concordance index (C-index) (23); (2) calibration curves, they were generated to compare the predicted *vs.* actual survival; (3) reclassification, the improvement of usefulness added by the radiomics signature was quantified by net reclassification improvement (NRI) (24); (4) clinical usefulness, it was determined by decision curve analysis (DCA) (25). In addition, the goodness-of-fit of all the models were assessed by the likelihood ratio test and BIC.

### Subgroup Analyses Based on Ultrasound Machines

To investigate whether different sonographic platforms affect the performance of radiomics signature for DFS prediction, we repeated Kaplan–Meier survival analysis in patients examined at GE healthcare and Mindray US systems, which were the most frequently used machines in this study.

### Statistical Analysis

Python software (Python Language Reference, version 3.6.9. Available at http://www.python.org) and R statistical software (version 4.0.0; R Foundation for Statistical Computing, Vienna, Austria) were used for all the statistical analyses. Chi-squared or Fisher’s exact test and Mann–Whitney U test were used to assess differences in distributions for categorical variables and continuous variables, respectively. The “lifelines” package was used for Kaplan–Meier survival analysis, log-rank test, and Cox regression. The “rms” package was used for the nomogram construction and calibration. NRI was calculated by “survIDINRI” package. The “rmda” package was used for DCA. A bilateral *P* value < 0.05 was considered significant.

## Results

### DFS and Clinicopathological Characteristics

The median period of follow-up was 48.99 (interquartile range [IQR], 44.42–62.98) months. Events occurred in 80 patients (80/620, 12.90%), including: 40 distant metastases; 17 locoregional recurrences; 12 locoregional recurrences and distant metastases; seven contralateral breast cancer (six invasive breast cancer and one DCIS); and four breast cancer-related death. The median DFS was 22.29 (IQR, 14.52–33.84) months. The comparison of the clinicopathological characteristics in the training and validation cohorts showed no significantly differences (**Table 1**).

**Table 1:** Characteristics of patients in the training and validation cohorts.

Characteristics	Training cohort (n = 372)	Validation cohort (n = 248)	*P-*value
Age, (years)^a^	49.10 ± 10.46	50.41 ± 10.76	0.094
Menopausal status Premenopausal Menopause	238 (63.98) 134 (36.02)	159 (64.11) 89 (35.89)	0.973
History of risk factors for breast cancer^b^ No Yes	359 (96.51) 13 (3.49)	240 (96.77) 8 (3.23)	0.856
Pathologic tumor size (cm)^a^	2.62 ± 1.40	2.51 ± 1.24	0.416
Molecular subtype Luminal A Luminal B HER2-enriched Triple-negative	76 (20.43) 204 (54.84) 55 (14.78) 37 (9.95)	46 (18.55) 130 (52.42) 39 (15.73) 33 (13.31)	0.571
TNM stage I II III	90 (24.19) 196 (52.69) 86 (23.12)	78 (31.45) 116 (46.77) 54 (21.77)	0.132
T stage 1 2 3 4	159 (42.74) 193 (51.88) 15 (4.03) 5 (1.34)	116 (46.77) 123 (49.60) 8 (3.23) 1 (0.40)	0.434^d^
N stage 0 1 2 3	178 (47.85) 113 (30.38) 53 (14.25) 28 (7.53)	126 (50.81) 73 (29.44) 34 (13.71) 15 (6.05)	0.847
ER status Negative Positive	96 (25.81) 276 (74.19)	74 (29.84) 174 (70.16)	0.270
PR status Negative Positive	113 (30.38) 259 (69.62)	94 (37.90) 154 (62.10)	0.052
HER2 status Negative Positive	255 (68.55) 117 (31.45)	163 (65.73) 85 (34.27)	0.463
Ki-67 status >14% ≤14%	292 (78.49) 80 (21.51)	196 (79.03) 52 (20.97)	0.873
Lymphovascular invasion Absent Present	239 (64.25) 133 (35.75)	159 (64.11) 89 (35.89)	0.973
Invasion of nerves Absent Present	305 (81.99) 67 (18.01)	206 (83.06) 42 (16.94)	0.730
Associated ductal carcinoma in situ Absent Present	274 (73.66) 98 (26.34)	180 (72.58) 68 (27.42)	0.767
Multifocal/multicentric disease No Yes	361 (97.04) 11 (2.96)	240 (96.77) 8 (3.23)	0.849
Histology type invasive ductal carcinoma invasive lobular carcinoma Others^c^	346 (93.01) 12 (3.23) 14 (3.76)	227 (91.53) 9 (3.63) 12 (4.84)	0.772
Type of surgery Mastectomy breast conservation surgery	322 (86.56) 50 (13.44)	222 (89.52) 26 (10.48)	0.271
Adjuvant endocrine therapy No Yes	113 (30.38) 259 (69.62)	85 (34.27) 163 (65.73)	0.308
Adjuvant chemotherapy No Yes	75 (20.16) 297 (79.84)	53 (21.37) 195 (78.63)	0.715
Adjuvant radiation No Yes	239 (64.25) 133 (35.75)	156 (62.90) 92 (37.10)	0.733
Adjuvant targeted therapy No Yes	315 (84.68) 57 (15.32)	208 (83.87) 40 (16.13)	0.787

Unless stated otherwise, data are numbers of patients, with percentages in parentheses.

a Data represent mean ± standard deviations.

b History of risk factors for breast cancer include six patients with family history of breast cancer, 14 patients with benign breast disease history, one patient with breast lesion biopsy history.

c Other cancers include 13 mucinous carcinomas, five papillary carcinomas, three medullary carcinomas, two metaplastic carcinomas, one tubular carcinoma, one cribriform carcinoma, one apocrine carcinoma.

d P value is calculated after combining T3 and T4 as one group because more than 20% of the expected frequencies are less than 5.

### The Radiomics Signature Construction and Validation

The mean ICC based on twice feature extraction was 0.824 (range, 0.798–0.999), which means the high intra-observer agreement for the radiomics feature extraction. Thence, all findings were based on the first feature extraction.

Totally, 14 features were selected from 1209 features to build radiomics signature in the training cohort (**Table S2** and **Figure S4**) and only one of them could distinguish patients with different prognoses (**Figures S5**, **S6**). The radiomics signature showed moderate performance on DFS estimation both in the training (C-index, 0.714; 95% confidence interval [CI], 0.63–0.80) and validation (C-index, 0.632; 95% CI, 0.52–0.74) cohorts. Based on the cutoff (1.816) of Rad-score (**Figure S7**), patients with higher Rad-score (≥1.816) were divided into the high-risk group, whereas patients with lower Rad-score (<1.816) were divided into the low-risk group, and their characteristics are shown in **Table 2**.

**Table 2:** Characteristics of patients according to the risk group based on radiomics signature in the training and validation cohorts.

Characteristics	Training cohort (n = 372)			Validation cohort (n = 248)		
	High-risk (n = 54)	Low-risk (n = 318)	*P-*value	High-risk (n = 50)	Low-risk (n = 198)	*P-*value
Rad-score	2.09 ± 0.21	1.37 ± 0.28	<0.0001	2.12 ± 0.30	1.30 ± 0.41	<0.0001
Age, (years)^a^	50.83 ± 11.15	48.80 ± 10.31	0.220	51.34 ± 10.72	50.17 ± 10.76	0.513
Menopausal status Premenopausal Menopause	29 (53.70) 25 (46.30)	222 (69.81) 96 (30.19)	0.019	29 (58) 21 (42)	117 (59.09) 81 (40.91)	0.889
History of risk factors for breast cancer^b^ No Yes	51 (94.44) 3 (5.56)	308 (96.86) 10 (3.14)	0.623	47 (94) 3 (6)	193 (97.47) 5 (2.53)	0.427
Pathologic tumor size (cm)^a^	3.46 ± 1.48	2.48 ± 1.33	<0.0001	3.14 ± 1.36	2.35 ± 1.15	0.00001
Molecular subtype Luminal A Luminal B HER2-enriched Triple-negative	6 (11.11) 39 (72.22) 7 (12.96) 2 (3.70)	72 (22.64) 169 (53.14) 44 (13.84) 33 (10.38)	0.043	5 (10) 23 (46) 15 (30) 7 (14)	39 (19.70) 103 (52.02) 28 (14.14) 28 (14.14)	0.042
TNM stage I II III	6 (11.11) 30 (55.56) 18 (33.33)	84 (26.42) 166 (52.20) 68 (21.38)	0.024	7 (14) 23 (46) 20 (40)	71 (35.86) 93 (46.97) 34 (17.17)	0.0004
T stage 1 2 3 4	9 (16.67) 37 (68.52) 5 (9.26) 3 (5.56)	150 (47.17) 156 (49.06) 10 (3.14) 2 (0.63)	<0.0001^d^	9 (18) 36 (72) 4 (8) 1 (2)	107 (54.04) 87 (43.94) 4 (2.02) 0 (0.00)	<0.0001^d^
N stage 0 1 2 3	17 (31.48) 22 (40.74) 7 (12.96) 8 (14.81)	161 (50.62) 91 (28.62) 46 (14.47) 20 (6.29)	0.016	21 (42) 12 (24) 11 (22) 6 (12)	105 (53.03) 61 (30.81) 23 (11.62) 9 (4.55)	0.039
ER status Negative Positive	17 (31.48) 37 (68.52)	79 (24.84) 239 (75.16)	0.303	16 (32) 34 (68)	58 (29.29) 140 (70.71)	0.709
PR status Negative Positive	19 (35.19) 35 (64.81)	94 (29.56) 224 (70.44)	0.406	21 (42) 29 (58)	73 (36.87) 125 (63.13)	0.504
HER2 status Negative Positive	32 (59.26) 22 (40.74)	223 (70.13) 95 (29.87)	0.112	32 (64) 18 (36)	131 (66.16) 67 (33.84)	0.774
Ki-67 status >14% ≤14%	49 (90.74) 5 (9.26)	243 (76.42) 75 (23.58)	0.018	42 (84) 8 (16)	154 (77.78) 44 (22.22)	0.334
Lymphovascular invasion Absent Present	25 (46.30) 29 (53.70)	214 (67.30) 104 (32.70)	0.003	23 (46) 27 (54)	136 (68.69) 62 (31.31)	0.003
Invasion of nerves Absent Present	43 (79.63) 11 (20.37)	262 (82.39) 56 (17.61)	0.626	40 (80) 10 (20)	166 (83.84) 32 (16.16)	0.518
Associated DCIS Absent Present	33 (61.11) 21 (38.89)	241 (75.79) 77 (24.21)	0.024	36 (72) 14 (28)	144 (72.73) 54 (27.27)	0.918
MF/MC disease No Yes	54 (100) 0 (0)	307 (96.54) 11 (3.46)	0.341	49 (98) 1 (2)	191 (96.46) 7 (3.54)	0.919
Histology type IDC ILC Others^c^	51 (94.44) 1 (1.85) 2 (3.70)	295 (92.77) 11 (3.46) 12 (3.77)	0.874^e^	48 (96) 1 (2) 1 (2)	179 (90.40) 8 (4.04) 11 (5.56)	0.324^e^
Type of surgery Mastectomy BCS	53 (98.15) 1 (1.85)	269 (84.59) 49 (15.41)	0.007	50 (100) 0 (0)	172 (86.87) 26 (13.13)	0.007
Adjuvant endocrine therapy No Yes	20 (37.04) 34 (62.96)	93 (29.25) 225 (70.75)	0.250	18 (36) 32 (64)	67 (33.84) 131 (66.16)	0.774
Adjuvant chemotherapy No Yes	11 (20.37) 43 (79.63)	64 (20.13) 254 (79.87)	0.967	8 (16) 42 (84)	45 (22.73) 153 (77.27)	0.300
Adjuvant radiation No Yes	34 (62.96) 20 (37.04)	205 (64.47) 113 (35.53)	0.831	32 (64) 18 (36)	124 (62.63) 74 (37.37)	0.857
Adjuvant targeted therapy No Yes	44 (81.48) 10 (18.52)	271 (85.22) 47 (14.78)	0.481	42 (84) 8 (16)	166 (83.84) 32 (16.16)	0.978

Unless stated otherwise, data are numbers of patients, with percentages in parentheses.

a Data represent mean ± standard deviations.

b History of risk factors for breast cancer include six patients with family history of breast cancer, 14 patients with benign breast disease history, one patient with breast lesion biopsy history.

c Other cancers include 13 mucinous carcinomas, five papillary carcinomas, three medullary carcinomas, two metaplastic carcinomas, one tubular carcinoma, one cribriform carcinoma, one apocrine carcinoma.

d P value is calculated after combining T3 and T4 as one group owing to the expected frequencies being <1.

e P value is calculated after combining ILC and Others as one group because more than 20% of the expected frequencies are less than 5.

Rad-score, radiomics score; DCIS, ductal carcinoma in situ; MF, multifocal; MC, multicentric; IDC, invasive ductal carcinoma; ILC, invasive lobular carcinoma; BCS, breast conservation surgery.

**Figure 1:**
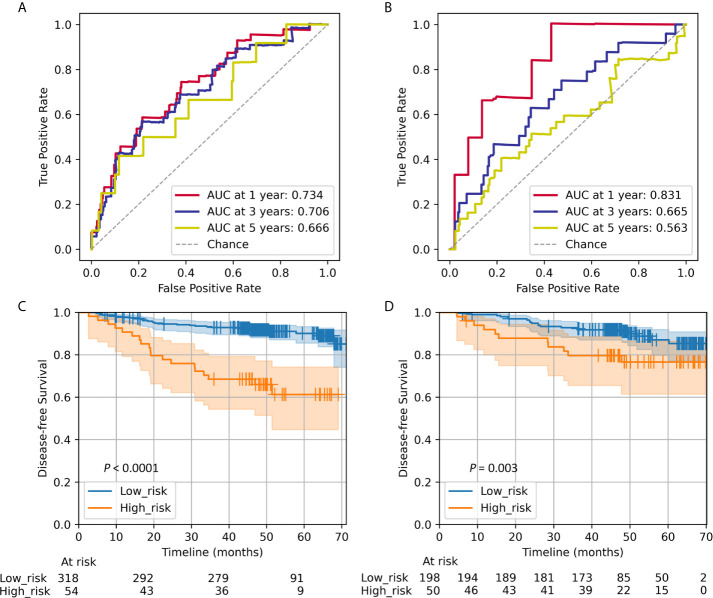
Radiomics score measured by time-dependent ROC curves and Kaplan–Meier survival curves in the training and validation cohorts. We used AUCs at 1, 3, and 5 years to assess prognostic accuracy in the training **(A)** and validation **(B)** cohorts. A significant association of the Rad-score with DFS was shown in the training **(C)** and validation **(D)** cohorts. We calculated *P* values using the log-rank test. Data are the AUC or *P*-value. ROC, receiver operator characteristics; AUC, area under the curve; DFS, disease-free survival.

The Rad-score prognostic accuracy determined by time-dependent receiver operator characteristics (ROC) curves and Kaplan–Meier survival curves are shown in **Figure 1**. The radiomics signature was significantly associated with DFS in the training (*P *< 0.0001) and validation (*P *= 0.003) cohorts. The 5-year DFS of the high- and low-risk groups were 61.27% and 90.10% in the training cohort and 76.60 and 87.07% in the validation cohort, respectively. The distribution of the Rad-score and DFS are shown in **Figures S8**–**S9**, patients with higher Rad-score were more likely to experience events.

**Figure 2:**
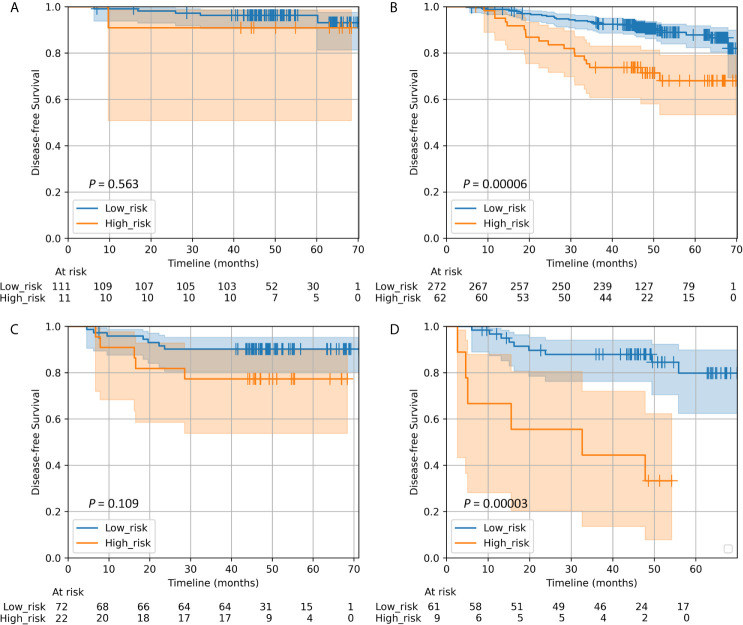
Kaplan–Meier survival curves of DFS according to the Rad-score classifier in subgroups within molecular subtypes of patients with invasive breast cancer in the whole cohort. **(A)** Luminal A (n = 122). **(B)** Luminal B (n = 334). **(C)** HER2-enriched (n = 94). **(D)** Triple-negative (n = 70). We calculated *P* values using the log-rank test. DFS, disease-free survival; Rad-score, radiomics score.

Results of stratified analysis based on molecular subtype are shown in **Figure 2**. The Rad-score successfully discriminate prognoses in luminal B (*P* = 0.00006) and triple-negative (*P* = 0.00003), but failed in either luminal A (*P* = 0.563) or HER2-enriched (*P* = 0.109). The radiomics signature remained a statistically and clinically predictor in most subgroups based on clinicopathological variables (**Figure S10**).

Both in the univariate (**Table S3**) and multivariable analyses (**Table 3**), the Rad-score was an independent predictor for DFS.

**Table 3:** Multivariate analysis of DFS in the training cohort.

Variable	Hazard ratio (95% CI)	*P-*value
Rad-score	3.95 (1.87–8.37)	0.0003
Lymphovascular invasion Absent Present	reference 2.19 (1.14–4.21)	/0.018
Molecular subtype Luminal A Luminal B HER2-enriched Triple-negative	reference 2.04 (0.61–6.81) 3.12 (0.80–12.18) 3.15 (0.75–12.43)	/0.247 0.031 0.038
N stage 0 1 2 3	reference 2.86 (1.21–6.73) 3.81 (1.46–9.97) 3.93 (1.15–12.31)	/0.016 0.006 0.007
T stage 1 2 3 4	Reference 1.46 (0.68–3.11) 2.90 (0.89–9.43) 0.80 (0.10–6.79)	/0.332 0.076 0.842

Rad-score, radiomics score; CI, confidence interval.

### The Additional Value of Radiomics Signature for DFS Prediction

**Figure 3:**
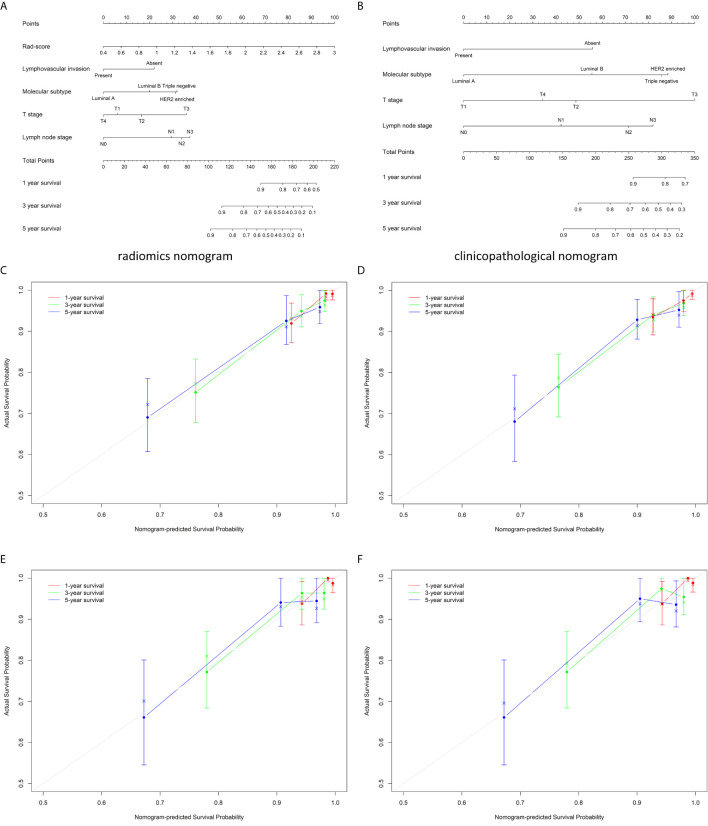
The developed radiomics nomogram **(A)** and clinicopathological nomogram **(B)** for DFS prediction in patients with invasive breast cancer, along with the calibration curves of these nomograms. The patient’s Rad-score is located on the Rad-score axis. To determine the number of points toward the probability of DFS the patient receives for her Rad-score, a line was drawn straight upward to the point axis, and this process was repeated for each variable. The points achieved for each of the risk factors was then summed. The final sum is located on the total point axis. To find the patient’s probability of DFS, a line was drawn straight down. Calibration curves of the radiomics nomogram in the training **(C)** and validation **(E)** cohorts, and those of the clinicopathological nomogram in the training **(D)** and validation **(F)** cohorts show the calibration of each model in terms of the agreement between the estimated and observed at 1-, 3-, and 5-year outcomes. Nomogram-estimated probability is plotted on the x-axis, and the actual survival probability is plotted on the y-axis. The diagonal gray line represents a perfect estimation by an ideal model, in which the estimated outcome perfectly corresponds to the actual outcome. The colored line represents the nomogram’s performance, a closer alignment of which with the diagonal dotted line represents a better estimation. DFS, disease-free survival; Rad-score, radiomics score.

**Figure 4:**
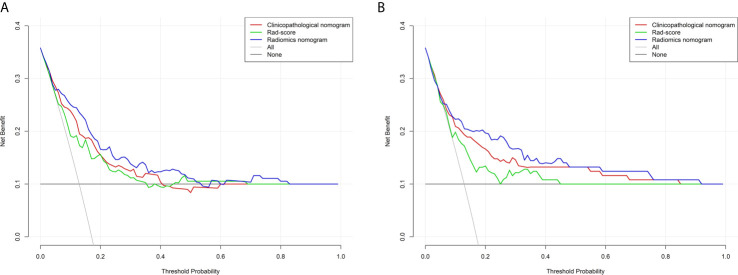
Decision curve analysis for each model in the training **(A)** and validation **(B)** cohorts. The y-axis measures the net benefit. The net benefit was calculated by summing the benefits (true positive results) and subtracting the harms (false positive results), weighing the latter by a factor related to the relative harm of an undetected cancer compared with the harm of unnecessary treatment. The radiomics model had the highest net benefit and simple strategies such as follow-up of all patients (gray line) or no patients (horizontal black line) across the full range of threshold probabilities at which a patient would choose to undergo imaging follow-up.

The estimation of the radiomics nomogram achieved a better agreement with actual observation than that of the clinicopathological nomogram (**Figure 3**). The radiomics nomogram yielded the highest C-index (0.801 and 0.796 in the training and validation cohorts, respectively), the highest log likelihood (−241.70), and the lowest BIC (502.75) (**Table 4**). Including the radiomics signature to the clinicopathological nomogram resulted improvement of classification accuracy for survival outcomes, with a total NRI of 0.147 in the validation cohort for 5-year DFS estimation (**Table S4**). Finally, the results of DCA demonstrated that the radiomics nomogram was superior than the clinicopathological nomogram in terms of clinical usefulness both in the training and validation cohorts (**Figure 4**).

**Table 4:** Performance of models.

Model	C-index (95%CI)		BIC	Log likelihood	*P-*value
	Training cohort	Validation cohort			
Radiomics signature	0.714 (0.63–0.80)	0.632 (0.52–0.74)	521.80	−258.97	<0.0001^a^
Clinicopathological nomogram	0.771 (0.69–0.85)	0.761 (0.66–0.86)	511.42	−247.97	<0.001^b^
Radiomics nomogram	0.801 (0.72–0.88)	0.796 (0.70–0.89)	502.75	−241.70	/

a The likelihood ratio test was performed between the radiomics signature and the radiomics nomogram.

b The likelihood ratio test was performed between the clinicopathological nomogram and the radiomics nomogram.

C-index, concordance index; CI, confidence interval.

### Subgroup Analyses Based on Ultrasound Machines

As shown in **Figure S11**, higher Rad-scores were significantly associated with worse DFS in the GE subgroup (*P *= 0.0001), but not in the Mindray subgroup (*P *= 0.055). Patients with higher Rad-scores experienced worse DFS than patients with lower Rad-scores in both the subgroups. Based on the cutoff (1.816) of the Rad-score, these patient characteristics based on risk group are shown in **Table S5**.

## Discussion

To our knowledge, this study has developed the first US radiomics features for DFS prediction of invasive breast cancer. We showed that the US radiomics signature was an independent factor in predicting DFS and confirmed its additional value added to the clinicopathological predictors.

The present radiomics signature comprised 14 features, including two 2D shape-based features, seven texture features, and five wavelet features. On the one hand, shape-based features reflect the shape and morphology of the tumor. Being consistent with a previous study which selected surface to volume ratio (SVR) to estimate breast cancer DFS (17), we selected PerimeterSurfaceRatio feature (the 2D form of SVR) as one of the 14 features. On the other hand, texture analysis is a suitable way to assess tumor heterogeneity (26), and different texture features are defined differently to depict specific aspects of tumor textural heterogeneity and thus may provide complementary information of tumor characteristics. Most texture and wavelet features selected in the present study could describe characteristics of breast cancer in previous study (12). Thus, the multiple-feature-based radiomics signature constructed in our study could likely be an important prognostic factor with the information of tumor heterogeneity.

In the following analyses, the radiomics signature showed moderate performance on DFS prediction and successfully stratified patients into different groups according to the results of risk stratification, though there was only one selected feature could stratify the risk of DFS. These findings were similar to a previous study of lung cancer which demonstrated that no individual feature could classify patients at different risk of recurrence, except for radiomics signature (27). Therefore, the radiomics signature, taking the interactions between different features into account, could better reflect the heterogeneity of tumor and is thus related to the outcome of patient, improving the accuracy of DFS assessment.

In the subsequently univariate, multivariate, and stratified analyses, the present US radiomics signature was an independent predictor, indicating the strong association between the radiomics signature and DFS. Patients at high-risk group experienced worse DFS than those at low-risk group, implying that patients at high risk of DFS might need more intensive treatment and follow-up to improve DFS, whereas treatment for low-risk patients could be attenuated appropriately. Consequently, our results would provide valuable information for clinicians to develop personalized treatment accurately based on the specific clinicopathological factors and radiomics signature for invasive breast cancer.

The Kaplan-Meier analyses performed by molecular subtype showed that only differences of DFS in luminal B and triple-negative subgroups were statistically significant. This suggested that the ability of radiomics signature to assess DFS for invasive breast cancer vary by molecular subtype, which was similar to an earlier MRI-based study (28). This also highlighted the fact that breast cancer is a heterogeneous tumor wherein every subtype has its unique characteristics and prognosis. Perhaps a specific radiomics signature for each molecular subtype would predict DFS better in invasive breast cancer and hence, further studies are needed to confirm this speculation.

Furthermore, we confirmed the additional value of radiomics signature to the clinicopathological predictors for DFS prediction. The single predictor is not enough to assess the probability of prognosis, whereas nomogram has the ability to integrate multiple factors. We constructed a radiomics nomogram in a step-wise manner based on BIC, achieving better performance compared to the clinicopathological nomogram, with a better calibration, positive NRI and higher C-index. Finally, the radiomics nomogram performed better than the clinicopathological nomogram in term of clinical usefulness, which confirmed the additional value of the radiomics signature for personalized DFS prediction in patients with invasive breast cancer simultaneously.

Finally, we analyzed whether different sonographic platforms affect the performance of radiomics signature and radiomics signature showed significant only in the GE subgroup. We think this may be related to small sample size of the Mindray subgroup (n = 121). Small sample size generally affects the performance of radiomics study (29). Taking small sample size into consideration, radiomics signature shows significant association with DFS in the Mindray subgroup when relax the significant *P* value to 0.1. Furthermore, the significant clinicopathological variables (tumor size, T stage, N stage, TNM stage and LVI) showed consistent in the GE and Mindray subgroups according to risk group based on radiomics signature. However, the probability of the dependency of radiomics signature on the type of US machine could not be entirely rule out and further studies with larger data are needed to reveal the truth of this problem.

Our study has some limitations. First, we could not control the operator dependency or scanning parameters in collecting US images, which is an inevitable issue. So, we used Z-score normalization to minimize the influence of contrast and brightness variation before feature extraction for each patient. Second, radiomics signature showed dependency on the type of US machine in present study. Third, this study had a relatively short follow-up period (median follow-up, 48.99 months) and no independent validation. Thus, further studies with a longer follow-up, independent data and larger sample size are needed to resolve these issues.

In summary, the US radiomics signature is a potential imaging predictor for risk stratification of DFS, the radiomics nomogram holds promise to serve as a noninvasive tool to assist clinicians in accurately developing personalized treatment for patients with invasive breast cancer.

## Data Availability Statement

The raw data supporting the conclusions of this article will be made available by the authors, without undue reservation.

## Ethics Statement

This study has obtained the ethical approval from the institutional review board of Sun Yat-Sen University Cancer Center.

## Author Contributions

LozL and LiL: guarantor of the article. LozL, LiL, LX, HC, and XT: conception and design. LX, HC, XT, BC, XJ, and LizL: collection and assembly of data. LX, HC, XT, and YF: data analysis and interpretation. All authors contributed to the article and approved the submitted version.

## Funding

We acknowledge support from the Science and Technology Planning Project of Guangdong Province (2017B020226004), the Science and Technology Program of Guangzhou (201807010057, 201907010043), and the Health and Medical Collaborative Innovation Project of Guangzhou (201803010021), and the Youth Fund Project of Guangdong Basic and Applied Basic Research Fund Regional Joint Fund (2020A1515110939).

## Conflict of Interest

The authors declare that the research was conducted in the absence of any commercial or financial relationships that could be construed as a potential conflict of interest.
