# Supplementary material for: Ultrasound-Based Radiomics Analysis for Predicting Disease-Free Survival of Invasive Breast Cancer
Source: Front Oncol. 2021 Apr 29;11:621993. doi: 10.3389/fonc.2021.621993 (PMC8117589; doi:10.3389/fonc.2021.621993)
Supplement: Supplementary file 1 [file DataSheet_1.docx]

Supplementary Material

# Supplementary Methods

**Feature extraction**

In order to minimize the influence of contrast and brightness variation, Z-score normalization was applied for each patient prior to feature extraction. Four categories of radiomics features including first-order statistics features, two-dimensional (2D) shape-based features, texture features, and wavelet features were calculated automatically. First-order statistics features described the distribution of pixel intensities within the image region defined by the mask through commonly used and basic metrics. 2D shape-based features included descriptors of the 2D size and shape of the region-of-interest (ROI). Texture features were calculated based on the gray level co-occurrence matrix (GLCM), the gray level size zone matrix (GLSZM), the gray level run length matrix (GLRLM), the neighborhood gray tone difference matrix (NGTDM) and the gray level dependence matrix (GLDM). Wavelet features were extracted using wavelet filtering, which yields 4 decompositions per level (all possible combinations of applying either a high or a low pass filter in each of the two dimensions) for an image. Note that we used three fixed bin widths (10, 15 and 25) in parameter setting of the feature extraction, resulting amounts of bins were somewhat between 15 and 130, which showed good reproducibility and performance in literature for a fixed bin count (1). The detailed list of all the radiomics features is shown in Table S2. The detailed definitions and calculations of all the radiomics features can be found in the image biomarker standardization initiative (2). Totally, 1209 features were extracted for each patient.

**Feature selection and construction of radiomics signature**

Before feature selection, each radiomics feature was standardized by removing its mean and scaling to unit variance. A two-step feature selection was performed in the training cohort. First, Spearman correlation coefficients and Ward linkage method were used to measure the correlations among features. Features with distance ≤6 formed a flat cluster. For each flat cluster, only the feature with the smallest *P* value in the univariate Cox regression was considered in the following step. After the first step of feature selection, 173 low-correlated features were selected. Then, we adopted the least absolute shrinkage and selection operator (LASSO) method, a popular regularized machine learning method for regression of high-dimensional data, to select the most useful predictive features in the training cohort. This method uses an L1 norm penalization to obtain a sparse solution that retains a small amount of non-zero feature coefficients. The parameter of λ in the LASSO is a constant that constrain the amount of non-zero coefficient and larger λ results in a smaller amount of non-zero feature coefficients. We tuned λ by using 10-fold cross validation and selected the optimal λ via 1-SE (standard error) criteria, which means the optimal λ is the largest value for which the partial likelihood deviance is within one SE of the smallest value of partial likelihood deviance. Finally, λ=0.0330303 was selected and the regularization path was shown in Figure S4. The optimal λ resulted in 14 features with non-zero coefficients, including original_shape2D_Elongation_10; original_shape2D_PerimeterSurfaceRatio_10; original_glcm_ClusterShade_25; original_glcm_Idmn_25; original_glszm_LGLZE_25; original_glszm_SZN_25; original_glszm_SZNN_25; original_gldm_DV_25; original_ngtdm_Coarseness_10; wavelet-LH_glcm_ClusterProminence_25; wavelet-HL_glrlm_LRLGLE_25; wavelet-HL_ngtdm_Contrast_25; wavelet-HH_glcm_ClusterShade_15 and wavelet-HH_glszm_GLNN_15, with coefficients 2.27553957e-01; -1.61817395e+01; 1.71107460e-03; 1.26090865e-05; -1.59511429e+00; 7.91319275e-04; 1.29075920e+00; 2.07883381e-01; -1.97503377e-05; 3.46206094e-05; -3.53562257e-02; -2.51895777e-01; 2.44114997e-01 and 1.64115701e+00.

The radiomics signature was constructed, with a radiomics score (Rad-score) calculated by using the following formula: Rad-score=2.27553957e-01 * original_shape2D_Elongation_10 - 1.61817395e+01 * original_shape2D_PerimeterSurfaceRatio_10 + 1.71107460e-03 * original_glcm_ClusterShade_25 + 1.26090865e-05 * original_glcm_Idmn_25 - 1.59511429e+00 * original_glszm_LGLZE_25 + 7.91319275e-04 * original_glszm_SZN_25 + 1.29075920e+00 * original_glszm_SZNN_25 + 2.07883381e-01 * original_gldm_DV_25 - 1.97503377e-05 * original_ngtdm_Coarseness_10 + 3.46206094e-05 * wavelet-LH_glcm_ClusterProminence_25 - 3.53562257e-02 * wavelet-HL_glrlm_LRLGLE_25 - 2.51895777e-01 * wavelet-HL_ngtdm_Contrast_25 + 2.44114997e-01 * wavelet-HH_glcm_ClusterShade_15 + 1.64115701e+00 * wavelet-HH_glszm_GLNN_15. The optimal cutoff of the Rad-score, dividing the patients into high- and low-risk groups, was generated using X-tile (Figure S7). More details about X-tile can be found in (3).

# Supplementary References

# Tixier F, Le Rest CC, Hatt M, Albarghach N, Pradier O, Metges JP, et al. Intratumor heterogeneity characterized by textural features on baseline 18F-FDG PET images predicts response to concomitant radiochemotherapy in esophageal cancer. *J Nucl Med.* (2011) 52:369-78. doi: 10.2967/jnumed.110.082404

# Zwanenburg A, Vallières M, Abdalah MA, Aerts HJWL, Andrearczyk V, Apte A, et al. The Image Biomarker Standardization Initiative: Standardized Quantitative Radiomics for High-Throughput Image-based Phenotyping. *Radiology.* (2020) 295:328-338. doi: 10.1148/radiol.2020191145

# Camp RL, Dolled-Filhart M, Rimm DL. X-tile: a new bio-informatics tool for biomarker assessment and outcome-based cut-point optimization. *Clin Cancer Res.* (2004) 10:7252-7259. doi: 10.1158/1078-0432.CCR-04-0713

## Supplementary Figures


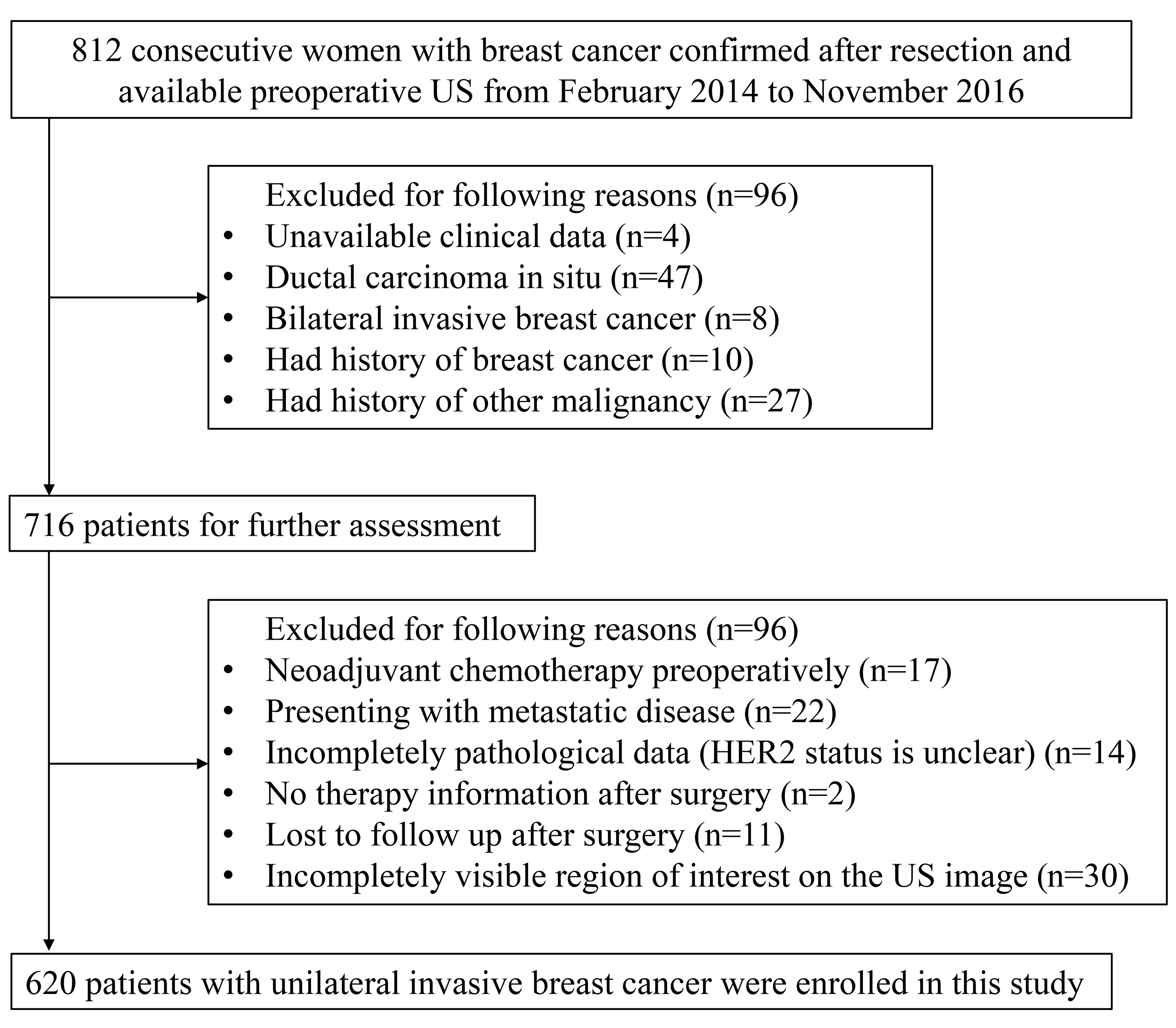


**Figure S1.** Patient recruitment protocol in this study.


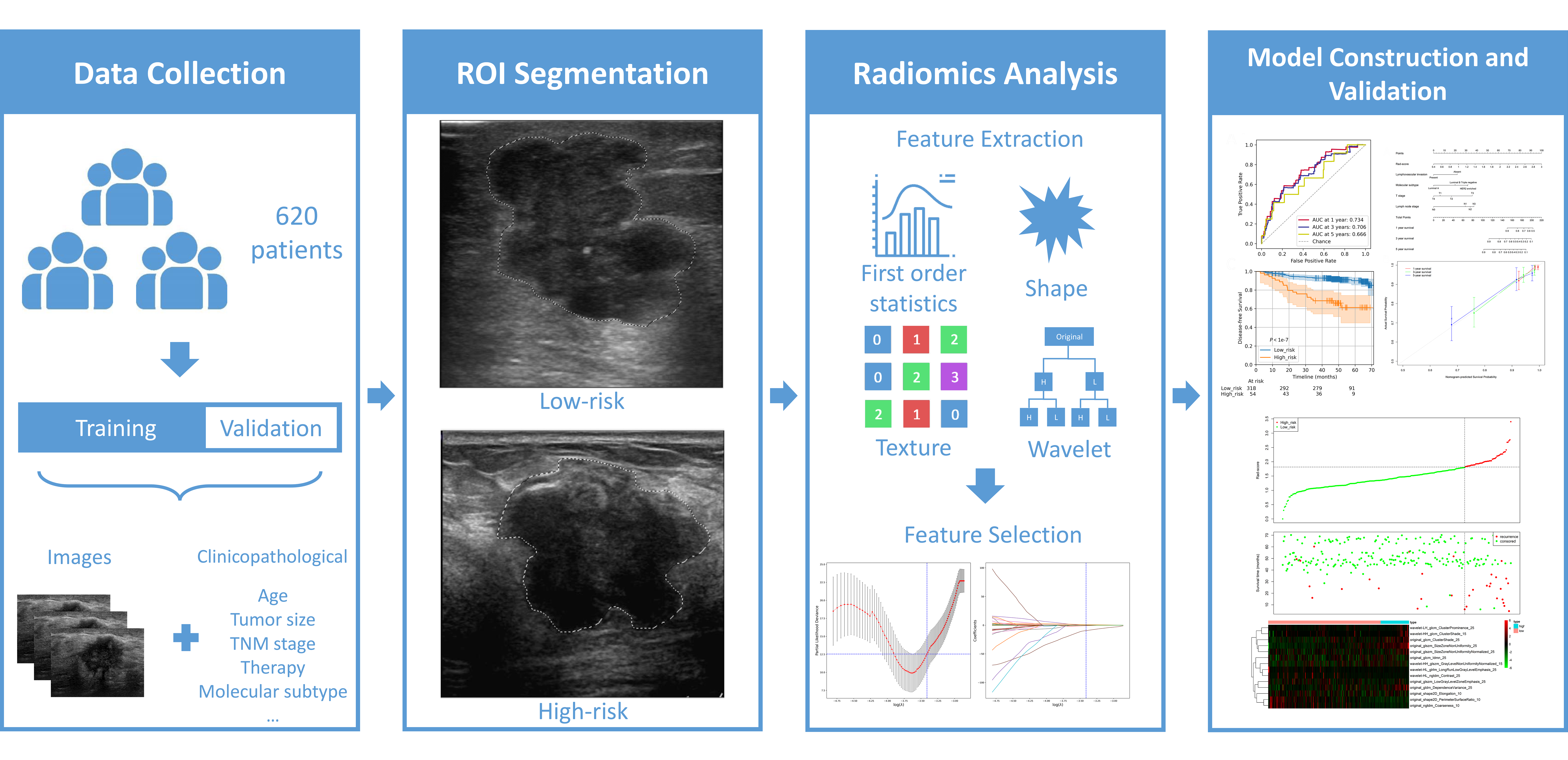


**Figure S2.** Radiomics workflow in this study. Firstly, total 620 patients with invasive breast cancer were enrolled, with their ultrasound images and clinicopathological data. Secondly, ROI segmentation was performed on the ultrasound images by radiologists. Then, four categories of radiomics features were extracted from within the defined ROI and the most useful predictive features were selected. Finally, the radiomics signature was constructed and validated, along with a Rad-score calculated. ROI, region-of-interest; Rad-score, radiomics score.


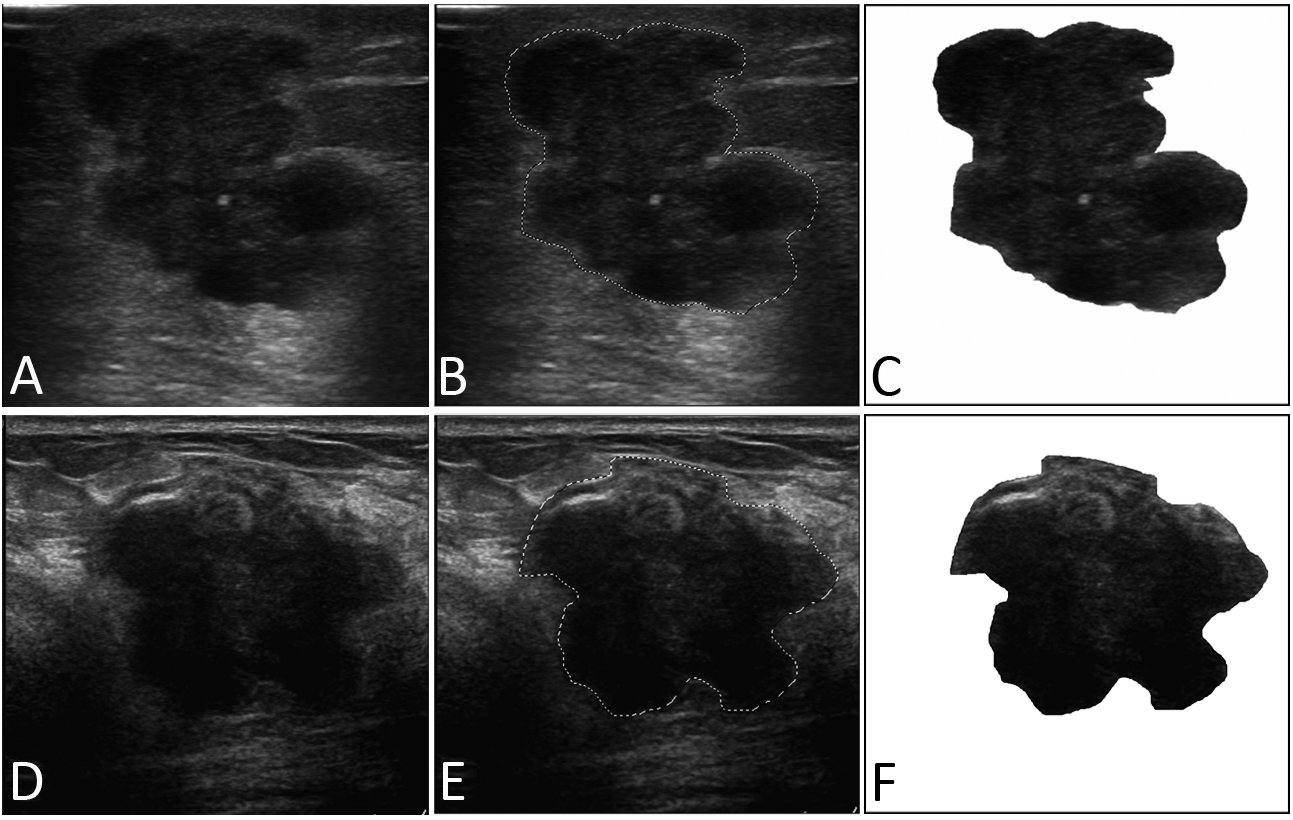


**Figure S3.** Two representative examples with defined ROI. **(A-C)** A 40-years old female patient with an irregular, heterogeneous hypoechoic lesion in the left breast, which was confirmed as a 3.8 cm-sized IDC by pathology. She was stratified into the low-risk group with a low Rad-score (1.672), and did not experience any events at the date of the last follow-up, her follow-up period was 50.0 months. (**D-F)** A 43-years old female patient with an irregular, heterogeneous hypoechoic lesion in the left breast, which was confirmed as a 4.0 cm-sized IDC by pathology. She was stratified into the high-risk group with a high Rad-score (1.993), and developed a contralateral triple-negative IDC 47.8 months later after surgery. ROI, region-of-interest; IDC, invasive ductal carcinoma; Rad-score, radiomics score.


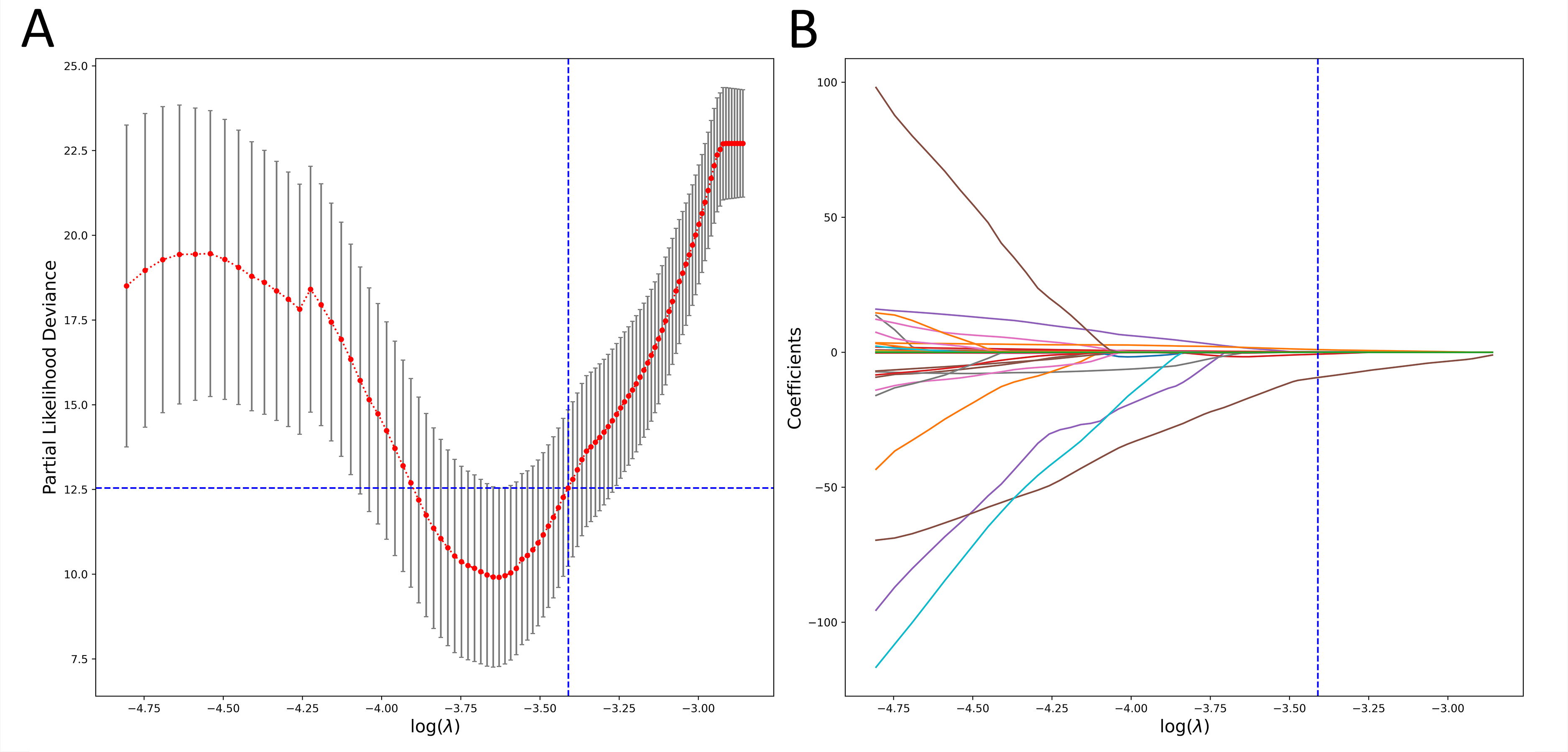


**Figure S4.** Radiomics feature selection using the least absolute shrinkage and selection operator (LASSO) Cox proportional hazards model. **(A)** Selection of the tuning parameter λ. The LASSO Cox regression model was used with penalty parameter tuning that was conducted by 10-fold cross-validation based on minimum criteria. The partial likelihood deviance curve was plotted versus log (λ). The dotted vertical line was plotted at the optimal λ value based on the minimum criteria and 1 standard error of the minimum criteria. The dotted horizontal was plotted at a partial likelihood deviance corresponding to the optimal λ. The optimal λ value of 0.0330303 with log (λ) = -3.4103299 was selected. **(B)** LASSO coefficient profiles of the 173 radiomics features. The dotted vertical line was plotted at the λ value of 0.0330303, resulting in 14 nonzero coefficients.


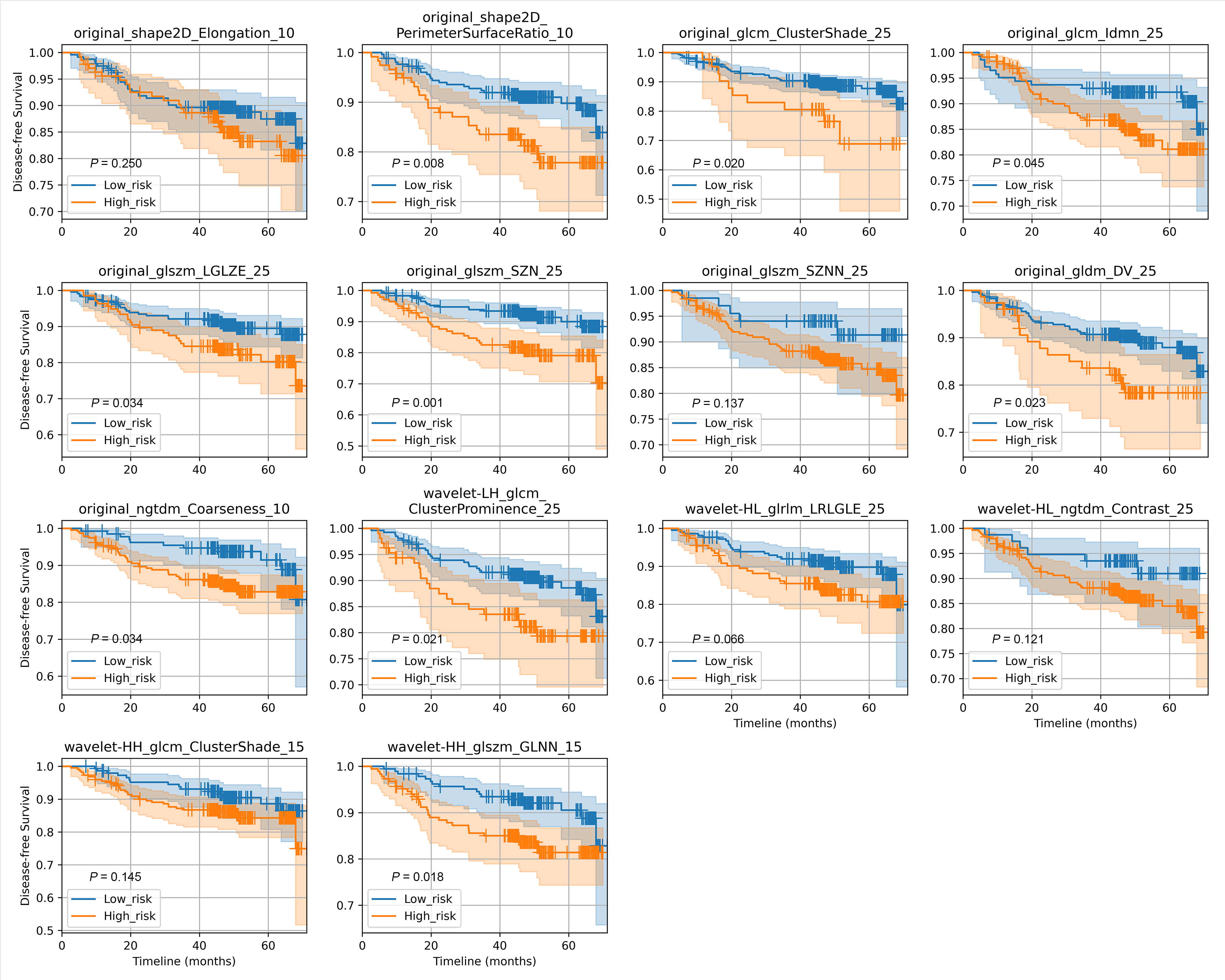


**Figure S5.** Kaplan–Meier survival curves of DFS for 372 patients with invasive breast cancer in the training cohort according to each of the selected features. *P* values were calculated by log-rank test. The cutoffs used to classify patients into high-risk and low-risk groups were generated by using X-tile in the training cohort and were as follows: original_shape2D_Elongation_10, 0.6680; original_shape2D_PerimeterSurfaceRatio_10, 0.0214; original_glcm_ClusterShade_25, 126.6433; original_glcm_Idmn_25, 0.9953; original_glszm_LGLZE_25, 0.0527; original_glszm_SZN_25, 288.5068; original_glszm_SZNN_25, 0.0922; original_gldm_DV_25, 4.8761 original_ngtdm_Coarseness_10, 0.0008; wavelet-LH_glcm_ClusterProminence_25, 5.3138; wavelet-HL_glrlm_LRLGLE_25, 0.4344; wavelet-HL_ngtdm_Contrast_25, 0.0145; wavelet-HH_glcm_ClusterShade_15, 0.0033; wavelet-HH_glszm_GLNN_15, 0.3113. 2D, two-dimensional; DFS, disease-free survival; GLCM, gray level co-occurrence matrix; GLSZM, gray level size zone matrix; GLRLM, gray level run length matrix; NGTDM, neighborhood gray tone difference matrix; GLDM, gray level dependence matrix.


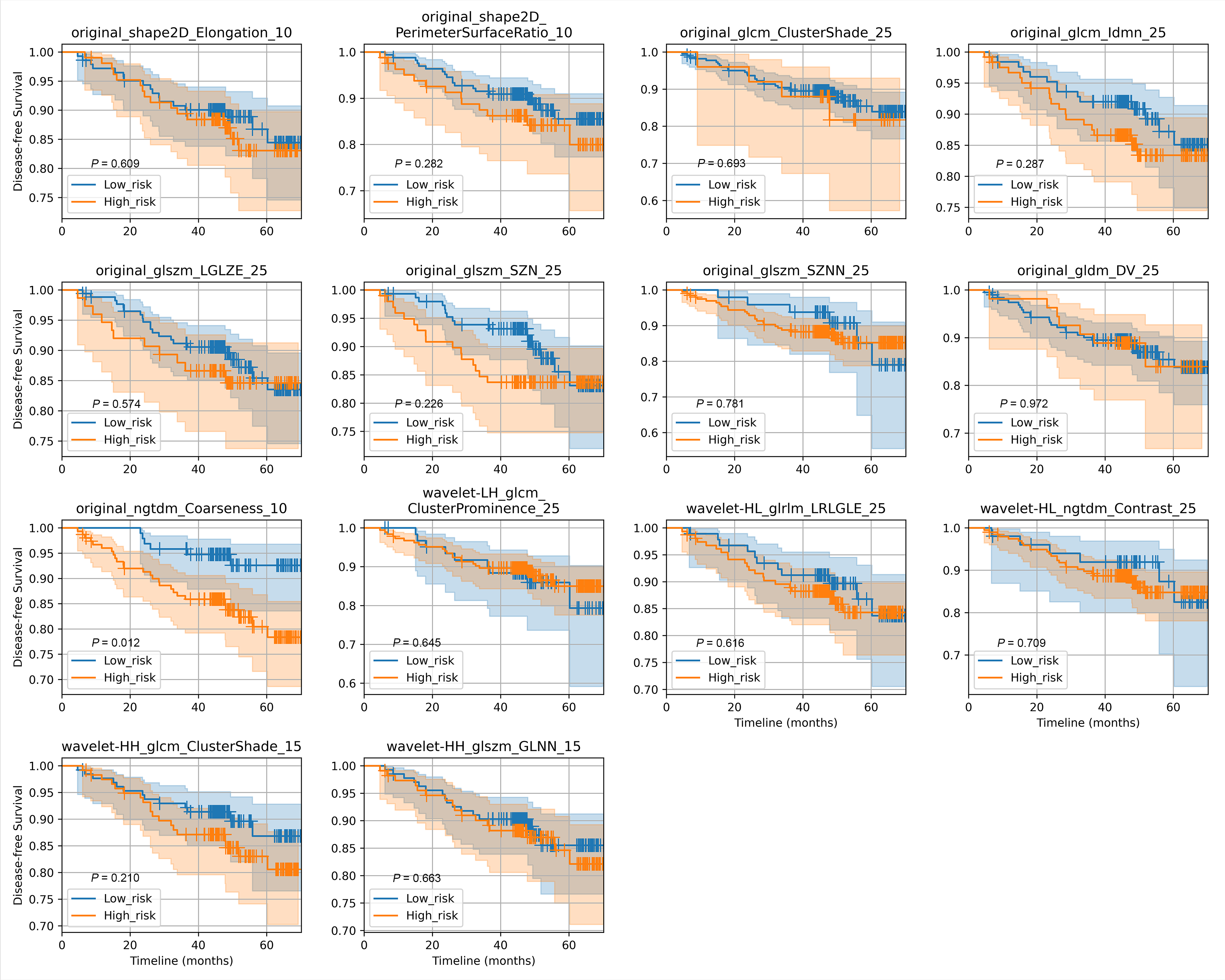


**Figure S6.** Kaplan–Meier survival curves of DFS for 248 patients with invasive breast cancer in the validation cohort according to each of the selected features. *P* values were calculated by log-rank test. The cutoffs used to classify patients into high-risk and low-risk groups were generated by using X-tile in the training cohort and were as follows: original_shape2D_Elongation_10, 0.6680; original_shape2D_PerimeterSurfaceRatio_10, 0.0214; original_glcm_ClusterShade_25, 126.6433; original_glcm_Idmn_25, 0.9953; original_glszm_LGLZE_25, 0.0527; original_glszm_SZN_25, 288.5068; original_glszm_SZNN_25, 0.0922; original_gldm_DV_25, 4.8761 original_ngtdm_Coarseness_10, 0.0008; wavelet-LH_glcm_ClusterProminence_25, 5.3138; wavelet-HL_glrlm_LRLGLE_25, 0.4344; wavelet-HL_ngtdm_Contrast_25, 0.0145; wavelet-HH_glcm_ClusterShade_15, 0.0033; wavelet-HH_glszm_GLNN_15, 0.3113. 2D, two-dimensional; DFS, disease-free survival; GLCM, gray level co-occurrence matrix; GLSZM, gray level size zone matrix; GLRLM, gray level run length matrix; NGTDM, neighborhood gray tone difference matrix; GLDM, gray level dependence matrix.


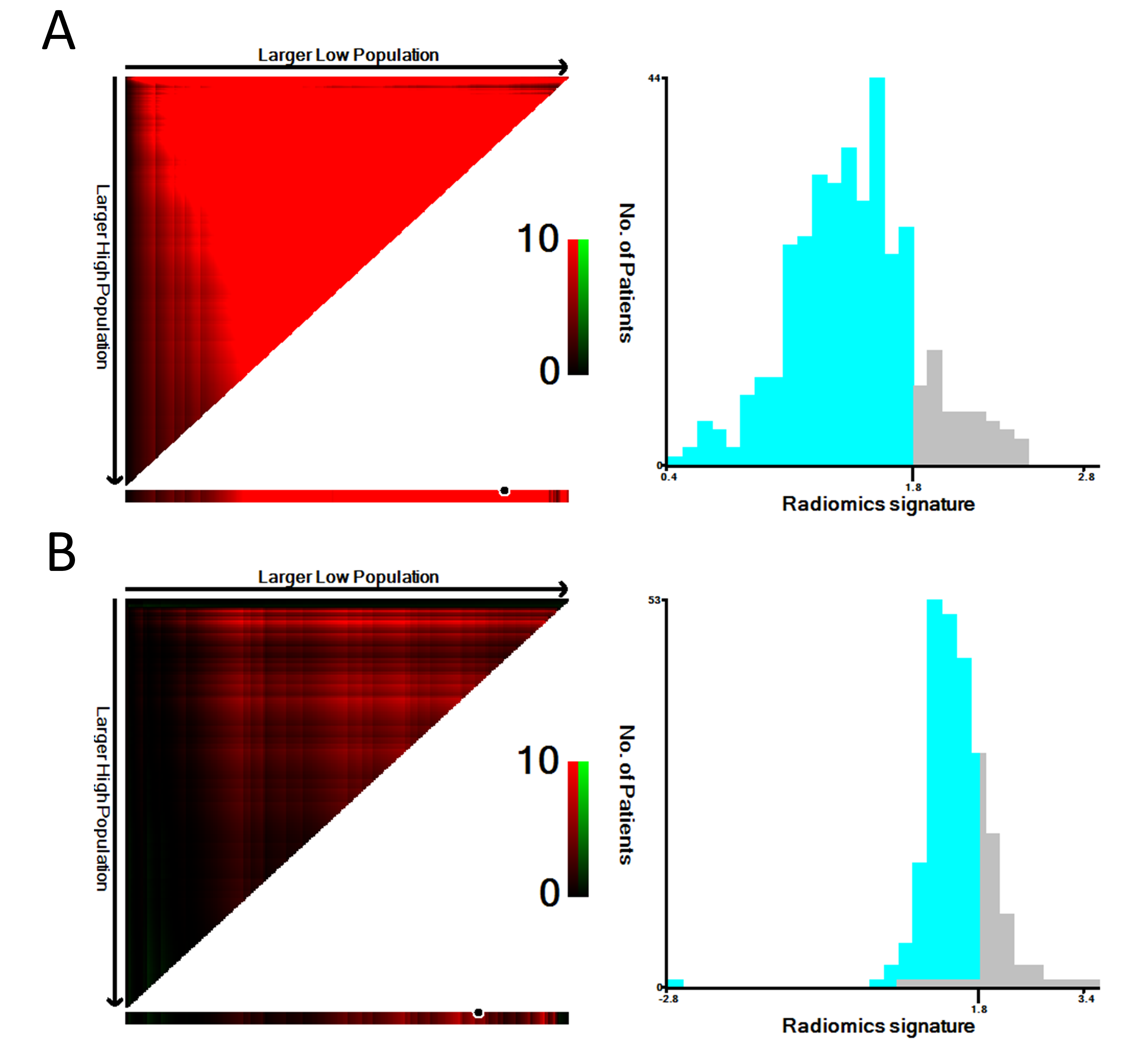


**Figure S7.** X-tile plots of the radiomics score associated with disease-free survival in the training cohort **(A)** and validation cohort **(B)**. The plots of the left panels show the log-rank values produced when dividing the dataset with one cutoff, producing high and low subsets (the rectangle grid at the bottom). The x-axis represents all potential cutoffs from low to high (left to right) that define a low subset, which increases in size in the direction from left to right. Red coloration of cutoff indicates an inverse correlation with survival, whereas green coloration represents direct associations. The optimal cutoff occurs at the brightest pixel (green or red). The cutoff highlighted by the small black circle in the left panels is shown on a histogram of the dataset (right panels).


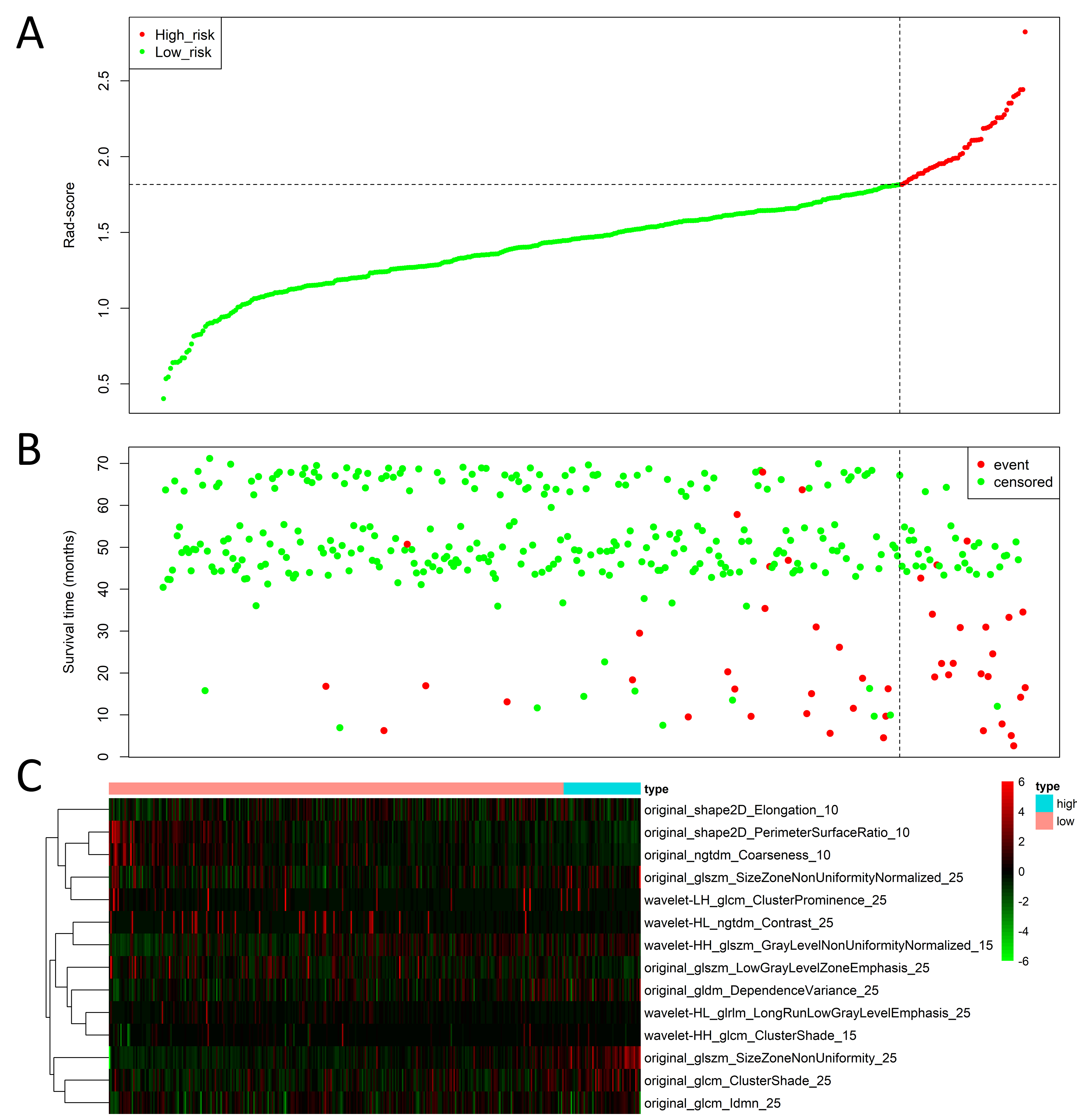


**Figure S8.** Rad-score analysis of 372 patients with invasive breast cancer in the training cohort. **(A)** Rad-score distribution: patients are sorted by Rad-scores in ascending order. **(B)** Survival time distribution: patients are sorted by Rad-scores in ascending order. **(C)** Color-gram of the expression profiles of 14 selected radiomics features: rows represent 14 radiomics features, and columns represent patients. Pink and blue type represent low-risk and high-risk group patients, respectively. Red represents inverse association between feature expression and DFS, whereas green represents direct association. Rad-scores, radiomics scores; DFS, disease-free survival.


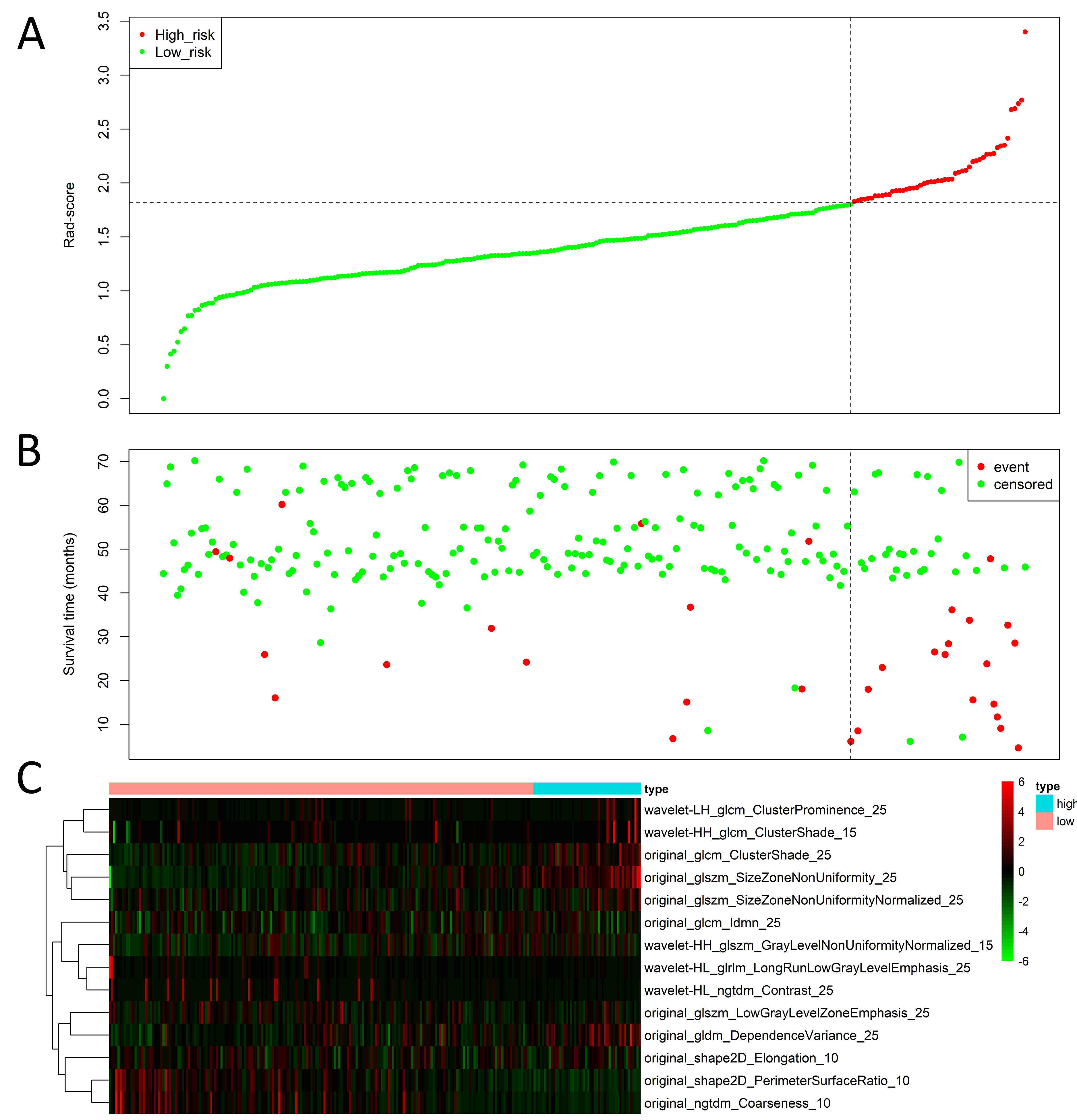


**Figure S9.** Rad-score analysis of 248 patients with invasive breast cancer in the validation cohort. **(A)** Rad-score distribution: patients are sorted by Rad-scores in ascending order. **(B)** Survival time distribution: patients are sorted by Rad-scores in ascending order. **(C)** Color-gram of the expression profiles of 14 selected radiomics features: rows represent 14 radiomics features, and columns represent patients. Pink and blue type represent low-risk and high-risk group patients, respectively. Red represents inverse association between feature expression and DFS, whereas green represents direct association. Rad-scores, radiomics scores; DFS, disease-free survival.


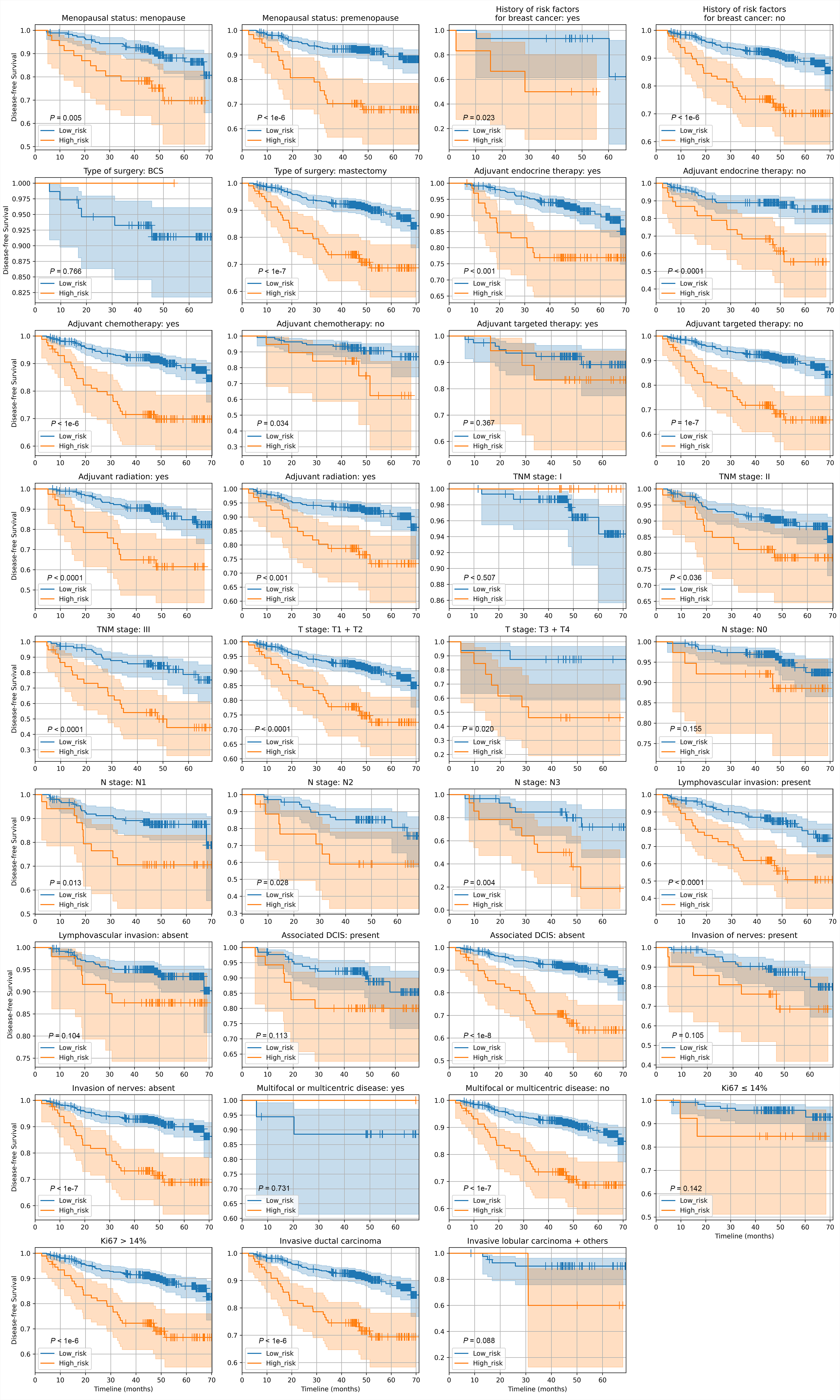


**Figure S10.** Kaplan–Meier survival curves of DFS for 620 patients with invasive breast cancer in the whole cohort according to the Rad-score classifier stratified by categorical clinicopathological variables. *P* values were calculated by log-rank test. DFS, disease-free survival; Rad-score, radiomics score; BCS, breast conservation surgery; DCIS, ductal carcinoma in situ.


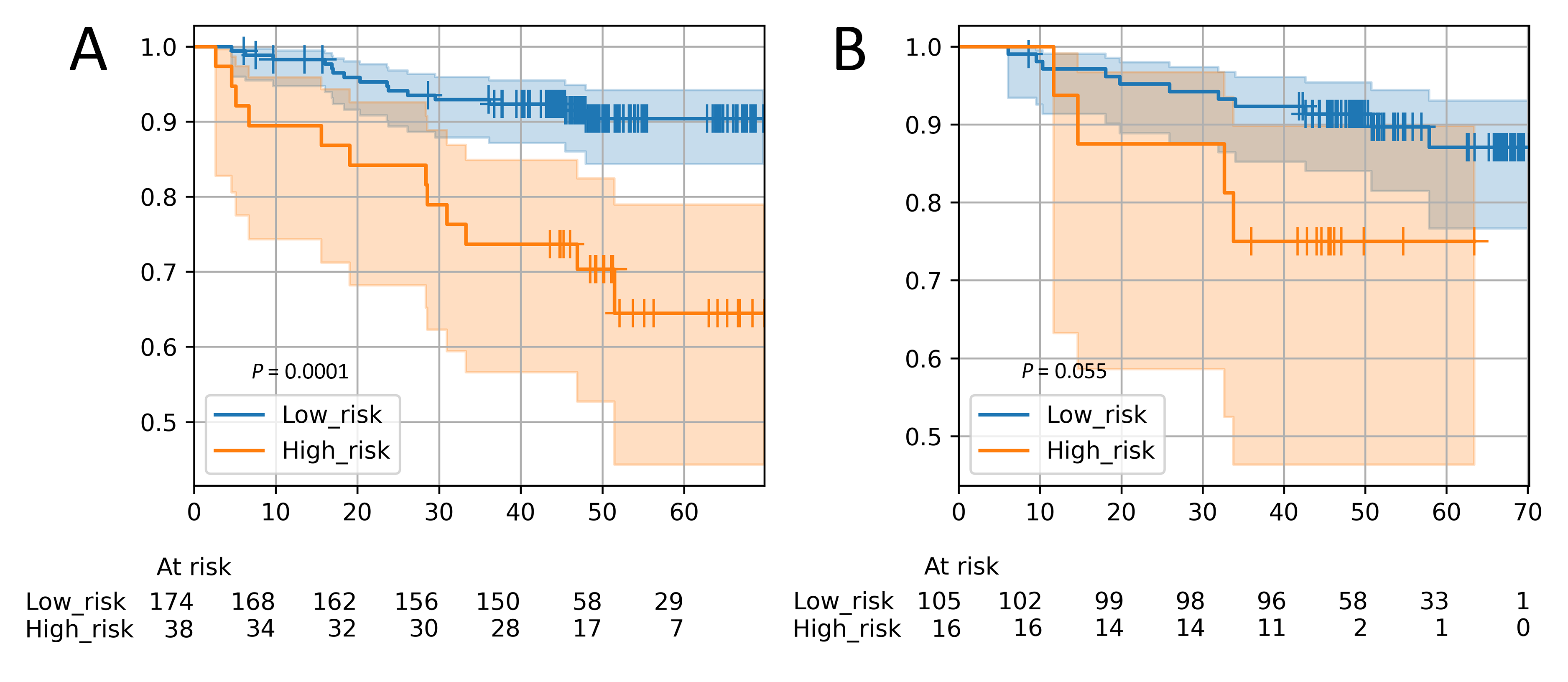


**Figure S11.** Kaplan–Meier survival curves of DFS for 212 patients with invasive breast cancer in the GE subgroup **(A)** and 121 patients with invasive breast cancer in the Mindray subgroup **(B)** according to the Rad-score classifier stratified by ultrasound machines. *P* values were calculated by log-rank test. DFS, disease-free survival; Rad-score, radiomics score.

## Supplementary Tables

**Table S1** Ultrasound machines used in this study.

| **Ultrasound machine** | **Training cohort**  **(n=372)** | **Validation cohort**  **(n=248)** |
| --- | --- | --- |
| ACUSON S2000 (Siemens, Erlangen, Germany) | 19 | 10 |
| MyLab D70 (Esaote, Italy) | 72 | 37 |
| LOQIG 7/S8/E9/vivid 7 (GE Healthcare, Wauwatosa, WI) | 128 | 84 |
| EUB-5500 (Hitachi Medical System, Tokyo, Japan) | 2 | 3 |
| DC-8 (Mindray ultrasound system, Shenzhen, China) | 70 | 51 |
| IU22 (Philips Medical Systems, best, the Netherlands) | 43 | 36 |
| Aixplorer (Supersonic, Aix-en-Provence, France) | 9 | 1 |
| Aplio XG 790 (Toshiba Medical Systems, Tokyo, Japan) | 27 | 26 |
| VINNO 70 (VINNO Medical system, Suzhou, China) | 2 | 0 |

**Table S2** List of radiomics features extracted in this study.

| **Feature class** | **Feature name** |
| --- | --- |
| First-order statistics features | Energy; Total Energy; Entropy; Minimum; 10th percentile; 90th percentile; Maximum; Mean; Median; Interquartile Range; Range; Mean Absolute Deviation (MAD); Robust Mean Absolute Deviation (rMAD); Root Mean Squared (RMS); Skewness; Kurtosis; Variance; Uniformity |
| two-dimensional (2D) shape-based features | Elongation; Major Axis Length; Maximum diameter; Mesh Surface; Minor Axis Length; Perimeter; Perimeter to Surface ratio; Pixel Surface; Sphericity |
| gray level co-occurrence matrix (GLCM) features | Autocorrelation; Joint Average; Cluster Prominence; Cluster Shade; Cluster Tendency; Contrast; Correlation; Difference Average; Difference Entropy; Difference Variance; Joint Energy; Joint Entropy; Informational Measure of Correlation (IMC) 1; Informational Measure of Correlation (IMC) 2; Inverse Difference Moment (IDM); Inverse Difference Moment Normalized (IDMN); Inverse Difference (ID); Inverse Difference Normalized (IDN); Inverse Variance; Maximum Probability; Sum Entropy; Sum of Squares |
| gray level size zone matrix (GLSZM) features | Gray Level Non-Uniformity (GLN); Gray Level Non-Uniformity Normalized (GLNN); Gray Level Variance (GLV); High Gray Level Zone Emphasis (HGLZE); Large Area Emphasis (LAE); Large Area High Gray Level Emphasis (LAHGLE); Large Area Low Gray Level Emphasis (LALGLE); Low Gray Level Zone Emphasis (LGLZE); Size-Zone Non-Uniformity (SZN); Size-Zone Non-Uniformity Normalized (SZNN); Small Area Emphasis (SAE); Small Area High Gray Level Emphasis (SAHGLE); Small Area Low Gray Level Emphasis (SALGLE); Zone Entropy (ZE); Zone Percentage (ZP); Zone Variance (ZV) |
| gray level run length matrix (GLRLM) features | Gray Level Non-Uniformity (GLN); Gray Level Non-Uniformity Normalized (GLNN); Gray Level Variance (GLV); High Gray Level Run Emphasis (HGLRE); Long Run Emphasis (LRE); Long Run High Gray Level Emphasis (LRHGLE); Long Run Low Gray Level Emphasis (LRLGLE); Low Gray Level Run Emphasis (LGLRE); Run Entropy (RE); Run Length Non-Uniformity (RLN); Run Length Non-Uniformity Normalized (RLNN); Run Percentage (RP); Run Variance (RV); Short Run Emphasis (SRE); Short Run High Gray Level Emphasis (SRHGLE); Short Run Low Gray Level Emphasis (SRLGLE) |
| neighborhood gray tone difference matrix (NGTDM) features | Busyness; Coarseness; Complexity; Contrast; Strength |
| gray level dependence matrix (GLDM) features | Dependence Entropy (DE); Dependence Non-Uniformity (DN); Dependence Non-Uniformity Normalized (DNN); Dependence Variance (DV); Gray Level Non-Uniformity (GLN); Gray Level Variance (GLV); High Gray Level Emphasis (HGLE); Large Dependence Emphasis (LDE); Large Dependence High Gray Level Emphasis (LDHGLE); Large Dependence Low Gray Level Emphasis (LDLGLE); Low Gray Level Emphasis (LGLE); Small Dependence Emphasis (SDE); Small Dependence High Gray Level Emphasis (SDHGLE); Small Dependence Low Gray Level Emphasis (SDLGLE) |

**Table S3** Univariate analysis of DFS in the training cohort.

| **Variable** | **Hazard ratio (95% CI)** | ***P*-value** |
| --- | --- | --- |
| Age**,** (years)^a^ | 1.01 (0.99–1.04) | 0.383 |
| Menopausal status  Premenopausal  Menopause | reference  1.11 (0.61–2.00) | 0.733 |
| History of risk factors for breast cancer^b^  No  Yes | reference  1.34 (0.32–5.52) | 0.686 |
| Pathologic tumor size (cm)^a^ | 1.29 (1.13–1.47) | 0.0002 |
| Molecular subtype  Luminal A  Luminal B  HER2-enriched  Triple-negative | reference  3.74 (1.14–12.24)  4.63 (1.25–17.10)  4.87 (1.22–19.49) | 0.030  0.022  0.015 |
| TNM stage  I  II  III | reference  11.97 (1.62–88.31)  26.15 (3.52–194.02) | 0.015  0.001 |
| T stage  1  2  3  4 | reference  2.71 (1.33–5.51)  6.66 (2.27–19.54)  3.87 (0.50–30.28) | 0.006  0.0005  0.197 |
| N stage  0  1  2  3 | reference  4.33 (1.91–9.83)  4.96 (2.00–12.35)  8.78 (3.39–22.79) | 0.0005  0.0006  0.000008 |
| Ki-67 status  ≤14%  >14% | reference  0.41 (0.16–1.03) | 0.0584 |
| Lymphovascular invasion  Absent  Present | reference  3.74 (2.06–6.77) | 0.00001 |
| Invasion of nerves  Absent  Present | reference  1.26 (0.63–2.53) | 0.517 |
| Associated DCIS  Absent  Present | reference  0.98 (0.51–1.88) | 0.945 |
| MF/MC disease  No  Yes | reference  1.49 (0.36–6.14) | 0.582 |
| Histology type  IDC  ILC  Others^c^ | reference  1.36 (0.33–5.62)  1.17 (0.28–4.82) | 0.669  0.830 |
| Type of surgery  Mastectomy  BCS | reference  0.77 (0.30–1.94) | 0.574 |
| Adjuvant endocrine therapy  No  Yes | reference  0.54 (0.30–0.95) | 0.034 |
| Adjuvant chemotherapy  No  Yes | reference  1.49 (0.67–3.33) | 0.329 |
| Adjuvant radiation  No  Yes | reference  1.43 (0.81–2.54) | 0.216 |
| Adjuvant targeted therapy  No  Yes | reference  0.96 (0.43–2.14) | 0.922 |
| Rad-score | 6.79 (3.38–13.62) | < 0.0001 |

Unless stated otherwise, data are numbers of patients, with percentages in parentheses. ^a^Data represent mean ± standard deviations. ^b^History of risk factors for breast cancer include six patients with family history of breast cancer, 14 patients with benign breast disease history, one patient with breast lesion biopsy history. ^c^Other cancers include 13 mucinous carcinomas, five papillary carcinomas, three medullary carcinomas, two metaplastic carcinomas, one tubular carcinoma, one cribriform carcinoma, one apocrine carcinoma. DCIS, ductal carcinoma in situ; MF, multifocal; MC, multicentric; IDC, invasive ductal carcinoma; ILC, invasive lobular carcinoma; BCS, breast conservation surgery; Rad-score, radiomics score; CI, confidence interval.

**Table S4** The Net reclassification improvement of adding the radiomics signature to the clinicopathological nomogram.

|  | **Training cohort** | |  | **Validation cohort** | |
| --- | --- | --- | --- | --- | --- |
| **Time** | **NRI (95% CI)** | ***P*-value** |  | **NRI (95% CI)** | ***P*-value** |
| 1 year | 0.184 (-0.16–0.44) | 0.340 |  | 0.331 (-0.05–0.66) | 0.647 |
| 3 years | 0.263 (0.03–0.42) | 0.036 |  | 0.127 (0.02–0.39) | 0.041 |
| 5 years | 0.242 (0.04–0.45) | 0.020 |  | 0.147 (0.08–0.42) | 0.035 |

NRI, Net reclassification improvement; CI, confidence interval.

**Table S5** Characteristics of patients who underwent US examination with GE healthcare and Mindray ultrasound system according to the risk group based on radiomics signature.

| **Characteristics** | **GE (n=212)** | | |  | | **Mindray (n=121)** | | |
| --- | --- | --- | --- | --- | --- | --- | --- | --- |
|  | **High-risk**  **(n=38)** | **Low-risk**  **(n=174)** | ***P*-value** | | **High-risk**  **(n=16)** | | **Low-risk**  **(n=105)** | ***P*-value** |
| Rad-score | 2.05 ± 0.20 | 1.40 ± 0.27 | < 0.0001 | | 2.10 ± 0.25 | | 1.30 ± 0.48 | < 0.0001 |
| Age, (years)^a^ | 51.08 ± 10.70 | 48.11 ± 9.52 | 0.121 | | 50.69 ± 12.59 | | 48.60 ± 11.35 | 0.566 |
| Menopausal status  Premenopausal  Menopause | 21 (55.26)  17 (44.74) | 119 (68.39)  55 (31.61) | 0.122 | | 8 (50.00)  8 (50.00) | | 75 (71.43)  30 (28.57) | 0.085 |
| History of risk factors for breast cancer^b^  No  Yes | 34 (89.47)  4 (10.53) | 169 (97.13)  5 (2.87) | 0.094 | | 16 (100.00)  0 (0.00) | | 102 (97.14)  3 (2.86) | 1.000 |
| Pathologic tumor size (cm)^a^ | 3.39 ± 1.54 | 2.31 ± 1.17 | < 0.0001 | | 3.54 ± 1.41 | | 2.57 ± 1.50 | 0.006 |
| Molecular subtype  Luminal A  Luminal B  HER2-enriched  Triple-negative | 3 (7.89)  24 (63.16)  6 (15.79)  5 (13.16) | 37 (21.26)  90 (51.72)  26 (14.94)  21 (12.07) | 0.289 | | 1 (6.25)  10 (62.50)  4 (25.00)  1 (6.25) | | 23 (21.90)  59 (56.19)  12 (11.43)  11 (10.48) | 0.612^d^ |
| TNM stage  I  II  III | 4 (10.53)  18 (47.37)  16 (42.11) | 54 (31.03)  88 (50.57)  32 (18.39) | 0.002 | | 1 (6.25)  8 (50.00)  7 (43.75) | | 32 (30.48)  54 (51.43)  19 (18.10) | 0.045^e^ |
| T stage  1  2  3  4 | 4 (10.53)  28 (73.68)  5 (13.16)  1 (2.63) | 94 (54.02)  76 (43.68)  4 (2.30)  0 (0) | < 0.0001^f^ | | 3 (18.75)  11 (68.75)  1 (6.25)  1 (6.25) | | 46 (43.81)  54 (51.43)  5 (4.76)  0 (0.00) | 0.057^g^ |
| N stage  0  1  2  3 | 13 (34.21)  13 (34.21)  6 (15.79)  6 (15.79) | 96 (55.17)  46 (26.44)  24 (13.79)  8 (4.60) | 0.025 | | 4 (25.00)  5 (31.25)  4 (25.00)  3 (18.75) | | 52 (49.52)  35 (33.33)  13 (12.38)  5 (4.76) | 0.037^h^ |
| ER status  Negative  Positive | 12 (31.58)  26 (68.42) | 48 (27.59)  126 (72.41) | 0.621 | | 5 (31.25)  11 (68.75) | | 24 (22.86)  81 (77.14) | 0.676 |
| PR status  Negative  Positive | 15 (39.47)  23 (60.53) | 62 (35.63)  112 (64.37) | 0.656 | | 6 (37.50)  10 (62.50) | | 27 (25.71)  78 (74.29) | 0.493 |
| HER2 status  Negative  Positive | 24 (63.16)  14 (36.84) | 111 (63.79)  63 (36.21) | 0.941 | | 9 (56.25)  7 (43.75) | | 72 (68.57)  33 (31.43) | 0.329 |
| Ki-67 status  >14%  ≤14% | 34 (89.47)  4 (10.53) | 133 (76.44)  41 (23.56) | 0.075 | | 15 (93.75)  1 (6.25) | | 80 (76.19)  25 (23.81) | 0.205 |
| Lymphovascular invasion  Absent  Present | 19 (50.00)  19 (50.00) | 124 (71.26)  50 (28.74) | 0.011 | | 5 (31.25)  11 (68.75) | | 74 (70.48)  31 (29.52) | 0.002 |
| Invasion of nerves  Absent  Present | 31 (81.58)  7 (18.42) | 145 (83.33)  29 (16.67) | 0.794 | | 11 (68.75)  5 (31.25) | | 87 (82.86)  18 (17.14) | 0.318 |
| Associated DCIS  Absent  Present | 26 (68.42)  12 (31.58) | 126 (72.41)  48 (27.59) | 0.621 | | 9 (56.25)  7 (43.75) | | 80 (76.19)  25 (23.81) | 0.167 |
| MF/MC disease  No  Yes | 38 (100.00)  0 (0.00) | 165 (94.83)  9 (5.17) | 0.323 | | 16 (100.00)  0 (0.00) | | 101 (96.19)  4 (3.81) | 1.00 |
| Histology type  IDC  ILC  Others^c^ | 35 (92.11)  2 (5.26)  1 (2.63) | 161 (92.53)  4 (2.30)  9 (5.17) | 0.803^i^ | | 16 (100.00)  0 (0.00)  0 (0.00) | | 96 (91.43)  5 (4.76)  4 (3.81) | 0.480^i^ |
| Type of surgery  Mastectomy  BCS | 38 (100.00)  0 (0.00) | 140 (80.46)  34 (19.54) | 0.003 | | 16 (100.00)  0 (0.00) | | 93 (88.57)  12 (11.43) | 0.329 |
| Adjuvant endocrine therapy  No  Yes | 17 (44.74)  21 (55.26) | 57 (32.76)  117 (67.24) | 0.161 | | 5 (31.25)  11 (68.75) | | 27 (25.71)  78 (74.29) | 0.870 |
| Adjuvant chemotherapy  No  Yes | 7 (18.42)  31 (81.58) | 41 (23.56)  133 (76.44) | 0.493 | | 1 (6.25)  15 (93.75) | | 18 (17.14)  87 (82.86) | 0.455 |
| Adjuvant radiation  No  Yes | 24 (63.16)  14 (36.84) | 112 (64.37)  62 (35.63) | 0.888 | | 7 (43.75)  9 (56.25) | | 70 (66.67)  35 (33.33) | 0.076 |
| Adjuvant targeted therapy  No  Yes | 32 (84.21)  6 (15.79) | 145 (83.33)  29 (16.67) | 0.895 | | 12 (75.00)  4 (25.00) | | 87 (82.86)  18 (17.14) | 0.681 |

Unless stated otherwise, data are numbers of patients, with percentages in parentheses. ^a^Data represent mean ± standard deviations. ^b^History of risk factors for breast cancer include two patients with family history of breast cancer, six patients with benign breast disease history, and one patient with breast lesion biopsy history in GE subgroup; one patient with family history of breast cancer and two patients with benign breast disease history in Mindray subgroup. ^c^Other cancers include six mucinous carcinomas, one papillary carcinoma, two medullary carcinomas and one metaplastic carcinoma in GE subgroup; three mucinous carcinomas and one cribriform carcinoma in Mindray subgroup. ^d^*P* value is calculated after combining luminal A and luminal B subtypes as one group, and combining HER2 enriched and triple negative subtypes into another group because more than 20% of the expected frequencies are less than 5. ^e^*P* value is calculated after combining TNM I and TNM II as one group because more than 20% of the expected frequencies are less than 5. ^f^*P* value is calculated after combining T3 and T4 as one group owing to the expected frequencies being <1. ^g^*P* value is calculated after combining T2, T3 and T4 as one group owing to the expected frequencies being <1. ^h^*P* value is calculated after combining N2 and N3 as one group because more than 20% of the expected frequencies are less than 5.  ^i^*P* value is calculated after combining ILC and Others as one group owing to the expected frequencies being <1. Rad-score, radiomics score; DCIS, ductal carcinoma in situ; MF, multifocal; MC, multicentric; IDC, invasive ductal carcinoma; ILC, invasive lobular carcinoma; BCS, breast conservation surgery.
